# Genome-Wide Identification and Expression Analysis of *ent-kaurene synthase-like* Gene Family Associated with Abiotic Stress in Rice

**DOI:** 10.3390/ijms25105513

**Published:** 2024-05-18

**Authors:** Yantong Teng, Yingwei Wang, Yutong Zhang, Qinyu Xie, Qinzong Zeng, Maohong Cai, Tao Chen

**Affiliations:** 1 Biotechnology Research Institute, Chinese Academy of Agricultural Sciences, Beijing 100081, China; 2 College of Life Sciences, Inner Mongolia University, Hohhot 010021, China; 3 Zhejiang Provincial Key Laboratory for Genetic Improvement and Quality Control of Medicinal Plants, College of Life and Environmental Science, Hangzhou Normal University, Hangzhou 311121, China

**Keywords:** *Oryza sativa*, KSL, GA, abiotic stress, plant height

## Abstract

Rice (*Oryza sativa*) is one of the most important crops for humans. The homologs of *ent*-kaurene synthase (KS) in rice, which are responsible for the biosynthesis of gibberellins and various phytoalexins, are identified by their distinct biochemical functions. However, the KS-Like (KSL) family’s potential functions related to hormone and abiotic stress in rice remain uncertain. Here, we identified the KSL family of 19 species by domain analysis and grouped 97 KSL family proteins into three categories. Collinearity analysis of *KSLs* among Poaceae indicated that the *KSL* gene may independently evolve and *OsKSL1* and *OsKSL4* likely play a significant role in the evolutionary process. Tissue expression analysis showed that two-thirds of *OsKSLs* were expressed in various tissues, whereas *OsKSL3* and *OsKSL5* were specifically expressed in the root and *OsKSL4* in the leaf. Based on the fact that *OsKSL2* participates in the biosynthesis of gibberellins and promoter analysis, we detected the gene expression profiles of *OsKSLs* under hormone treatments (GA, PAC, and ABA) and abiotic stresses (darkness and submergence). The qRT-PCR results demonstrated that *OsKSL1*, *OsKSL3*, and *OsKSL4* responded to all of the treatments, meaning that these three genes can be candidate genes for abiotic stress. Our results provide new insights into the function of the KSL family in rice growth and resistance to abiotic stress.

## 1. Introduction

*ent-kaurene synthase* (KS), one of the diterpene synthases (diTPS), is an enzyme that plays a vital role in the biosynthesis of *ent*-kaurene, a precursor of gibberellins [1,2,3]. The biosynthesis of *ent*-kaurene serves as a crucial initial step in gibberellin synthesis [4,5]. KS mutants commonly exhibit a dwarf phenotype, which can be rescued by the application of exogenous gibberellin (GA) [6,7]. Furthermore, the mutation of KS leads to a non-heading phenotype in plants [8,9]. Studies have also demonstrated that the expression of the *ent-kaurene synthase* gene can be up-regulated by blue light, influencing the avoidance response of protonemal growth in *Physcomitrella patens* [10,11]. *ent*-kaurene biosynthesis and gibberellin biosynthesis are prevalent in various organisms, including angiosperms, gymnosperms, spore plants, fungi, and bacteria [12,13]. The formation of *ent*-kaurene occurs through a two-step cyclization process of geranylgeranyl diphosphate, which involves the formation of an intermediate known as *ent*-copalyl diphosphate (*ent*-CDP) [14,15,16].

The pathway of *ent*-kaurene biosynthesis was initially elucidated using cell-free systems from *G. fujikuroi*. The two cyclization steps that convert geranylgeranyl diphosphate (GGDP) to *ent*-kaurene are catalyzed by bifunctional diTPS, which possesses two active sites [17,18,19,20]. The N-terminal active site domain harbors a conserved DXDD motif and catalyzes the protonation-initiated cyclization of GGDP to *ent*-CDP [21]. In the C-terminal domain, a conserved DDXXD motif is essential for the diphosphate ionization-initiated cyclization of *ent*-CDP to *ent*-kaurene [22].

In angiosperms and gymnosperms, the two consecutive cyclization reactions are catalyzed by two distinct monofunctional diterpene synthases: *ent*-copalyl diphosphate synthase (CPS, formerly *ent-kaurene synthase* A) and *ent-kaurene synthase* (KS, formerly *ent-kaurene synthase* B). CPS possesses the DXDD motif and exhibits *ent*-copalyl diphosphate synthase activity, while KS contains only the DDXXD motif and is responsible for synthase activity [23]. 

Beyond the involvement in gibberellin biosynthesis, *ent*-kaurene is an important intermediate in many specialized diterpenoid metabolic pathways, including the biosynthesis of various phytoalexins. Rice produces a variety of phytoalexins, including phytocassanes A-E, momilactones A and B, oryzalexins A-F, and oryzalexin S [24,25]. Many of them are related to *kaurene synthase-like* (KSL) family genes. Specifically, *OsKSL4*, *OsKSL7*, *OsKSL8*, and *OsKSL10* encode syn-pimara-7,15-diene synthase, *ent*-cassadiene synthase, stemar-13-ene synthase, and *ent*-sandaracopimaradiene synthase, respectively, contributing to the biosynthesis of labdane-related phytoalexins [26]. *OsKSL6* encodes *ent*-isokaurene synthase and is responsible for oryzadione biosynthesis [27]. In maize, *ZmKSL3* and *ZmKSL5* are proposed to be involved in diterpenoid phytoalexin biosynthesis due to their inducible expression patterns [28].

In this study, we identified and characterized 97 KSL proteins of 19 species belonging to evolutionary nodes or economic crops. In addition, gene duplication events among different species and syntenic analysis in rice were shown. *OsKSL* expression patterns in tissues and under various hormones and abiotic stresses were detected. Taken together, *OsKSL1*, *OsKSL3*, and *OsKSL4* can be used to explore more functions in rice development and growth. These findings serve not only to assign the function to a further important OsKSL family member, but also provide a theoretical basis for rice breeding.

## 2. Results

### 2.1. Identification of KSL Family Members in Major Crops

PLN02279 (from the Conserved Protein Domain database in the NCBI) represents the conserved domain of KS family proteins. The domain of Terpene_synth (PF01397) and Terpene_synth_C (PF03936) are included in PLN02279. Here, we selected crops for the study objects, including monocotyledonous crops such as *Oryza sativa* and *Zea mays*, and dicotyledonous crops such as *Brassica napus* and *Solanum tuberosum*. Importantly, the planting area of these species covers most of the crop area. Moreover, the model plant *Arabidopsis thaliana* and two evolutionary nodes *Selaginella moellendorfii* and *Phalaenopsis equestris* were included. Then, a total of 97 nonredundant KS proteins with conserved KS domains across 19 plant genomes were identified (Table S1). The distribution of these KS proteins across the different species is as follows: *Selaginella moellendorfii* (*Sm*) (4), *Oryza sativa* (*Os*) (9), *Zea mays* (*Zm*) (5), *Triticum aestivum* (*Ta*) (24), *Hordeum vulgare* (*Hv*) (5), *Sorghum bicolor* (*Sb*) (1), *Arabidopsis thaliana* (*At*) (2), *Brassica napus* (*Bn*) (4), *Phalaenopsis equestris* (*Pe*) (4), *Nicotiana tabacum* (*Nt*) (8), *Solanum lycopersicum* (*Sl*) (5), *Solanum tuberosum* (*St*) (3), *Lactuca sativa* (*Ls*) (3), *Helianthus annuus* (*Ha*) (6), *Glycine max* (*Gm*) (2), *Pisum sativum* (*Ps*) (3), *Vigna radiata* (*Vr*) (2), *Arachis hypogaea* (*Ah*) (2), and *Gossypium hirsutum* (*Gh*) (5). 

The identified genes were systematically renamed *KSL1* to *KSLn* and the basic information was analyzed and are summarized in Table 1. These characteristics include the number of amino acids, molecular weight, and isoelectric point. The number of amino acids in these proteins varies from 716 to 950, while their MW ranges from 81 to 106 kDa. The PI values range from 5.03 to 7.29, indicating the range of acidity or basicity of the proteins. These comprehensive measurements provide valuable insights into the diverse characteristics of the identified proteins.

### 2.2. Phylogenetic Analysis and Classification of the KSL Family

**Figure 1:**
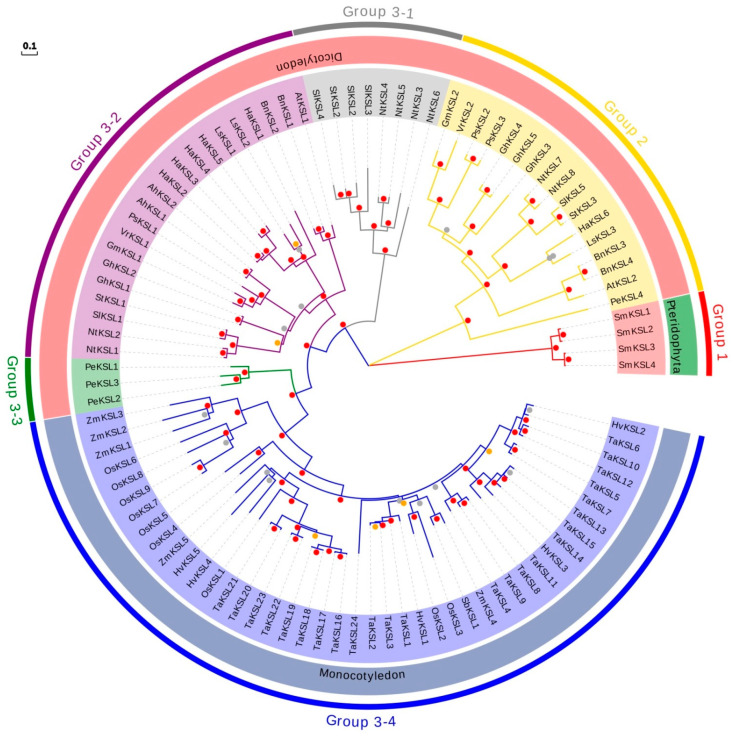
Phylogenetic analysis of the KSL family. Phylogenetic tree constructed by 97 proteins from 19 plant species. The 6 subfamilies of KSLs, including group 1, group 2, group 3-1, group 3-2, group 3-3, and group 3-4, are labeled with different colors. The 19 plant species are grouped into three major clades: Pteridophyta, dicotyledon, and monocotyledon, marked with different colors. Different colored points in the middle of the evolutionary tree branch represent different bootstrap values, red dots (80–100), gold dots (60–80), and gray dots (0–60).

To investigate the phylogenetic relationships among KSL family members in plants, a rooted maximum likelihood phylogenetic tree was generated with the 97 KSL proteins from the 19 species (Figure 1). The phylogenetic analysis revealed three distinct groups, which were strongly supported by a high-confidence bootstrap value greater than 95%, designated as group 1, group 2, and group 3. Furthermore, group 3 exhibited further subdivision into four subclasses, namely, group 3-1, group 3-2, group 3-3, and group 3-4.

The distribution of KSL proteins within the 19 species was analyzed and are summarized in Table 2. Among them, group 1 exclusively consisted of four KSL members from *Selaginella moellendorfii*, representing the Pteridophyta division. Group 2, group 3-1, group 3-2, and group 3-3 all belong to dicotyledon. In *Prunus equestris*, one KSL member was assigned to group 2, while three KSLs were categorized under group 3-3. For *Nicotiana tabacum*, *Solanum lycopersicum*, and *Solanum tuberosum*, known as Solanaceae plants, their KSLs were distributed among group 2, group 3-1, and group 3-2. Similarly, other dicotyledonous plants such as *Arabidopsis thaliana* and *Helianthus annuus* demonstrated KSL distribution in group 2 and group 3-2. In contrast, those in group 3-4 were exclusive to monocotyledon, and the KSLs of five monocotyledonous plants, including *Oryza sativa* and *Zea mays*, were categorized under this group. Generally, the distribution of KSL members among groups implied the evolutionary relationship of the KSL family.

### 2.3. Conserved Motif, Conserved Domain, and Gene Structure Analysis of KSL Family

**Figure 2:**
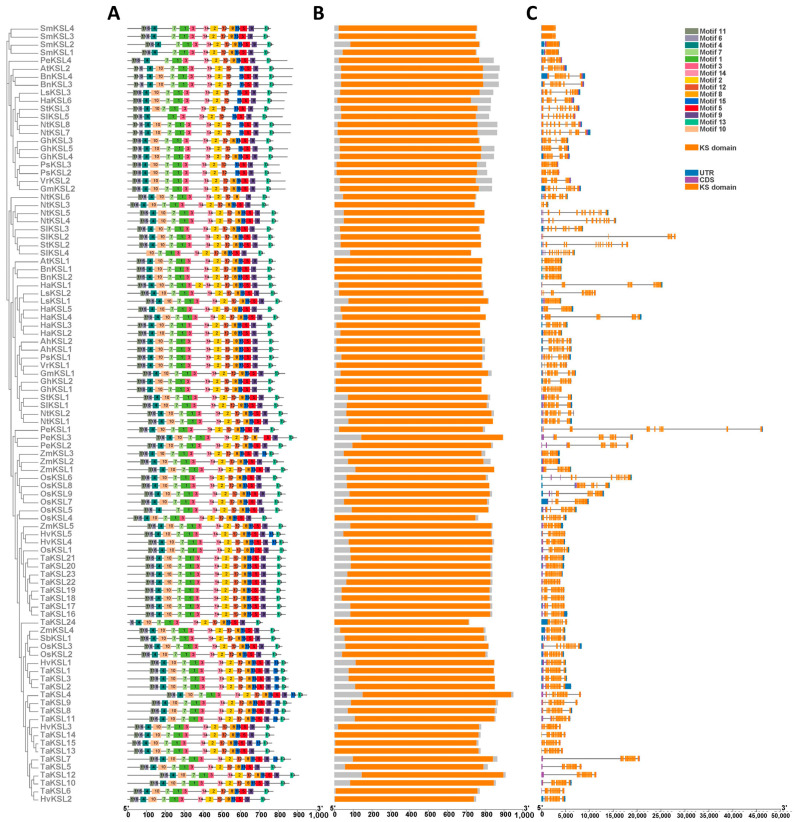
Phylogenetic relationships and structural features of 97 *KSL* genes in 19 species. (**A**) Motif composition: The diversity and distribution of conserved motifs in the KSL proteins are depicted as colored boxes, each representing a distinct motif, annotated with motif numbers for identification. (**B**) Domain architecture: The consistent presence of the KS (keto-synthase) domain within *KSL* genes is illustrated by orange boxes, while the gray boxes delineate regions outside the KS domain. (**C**) Gene structure: Exonic regions of *KSL* genes are represented by orange boxes for coding sequences and purple boxes for untranslated regions (UTRs), while intronic sequences are depicted by gray lines connecting exons.

To gain further insights into the motifs present in the KSL protein sequences, we executed the MEME online tool to identify and analyze 15 motifs in 97 KSL protein sequences (Figure S1). The composition and arrangement of the motifs are shown in Figure 2A. Although the motifs were generally similar, each of the three groups of KSLs, group 1, group 2, and group 3, exhibited distinct characteristics. Specifically, group 1 lacked motif 10 in all of its four KSLs, group 2 showed the absence of motif 8 in most of its KSLs, and group 3 generally contained all 15 motifs. These findings strongly suggested the existence of a significant relationship between motif composition differences and the functional divergence of KSL proteins within the three distinct groups. 

PLN02279 represents the characteristic structure of the KS (*ent*-kaur-16-ene synthase) superfamily according to the NCBI CDD database. As shown in Figure 2B, the KS domain was highly conserved among KSL members, with all 97 identified KSLs containing this structure. PLN02279 served as the primary functional structure within KSL proteins, encompassing the majority of their amino acid composition. However, at the protein domain level, group 2 differed from other groups in that there were some additional regions at the C terminal of the KSL protein.

The *KSL* gene structures were visualized based on the GFF3 files (Figure 2C). Although KSL members were conserved in protein structures, there was considerable diversity in terms of gene structures both within and between species. For example, some genes were notably shorter, measuring less than 5000 bp, while the longest gene, namely, *PeKSL1*, spanned over 45,000 bp. It is worth noting that these differences in gene length primarily stemmed from variations in intron content.

### 2.4. Analysis of Cis-Acting Elements in the Promoter Region of KSL Genes

**Figure 3:**
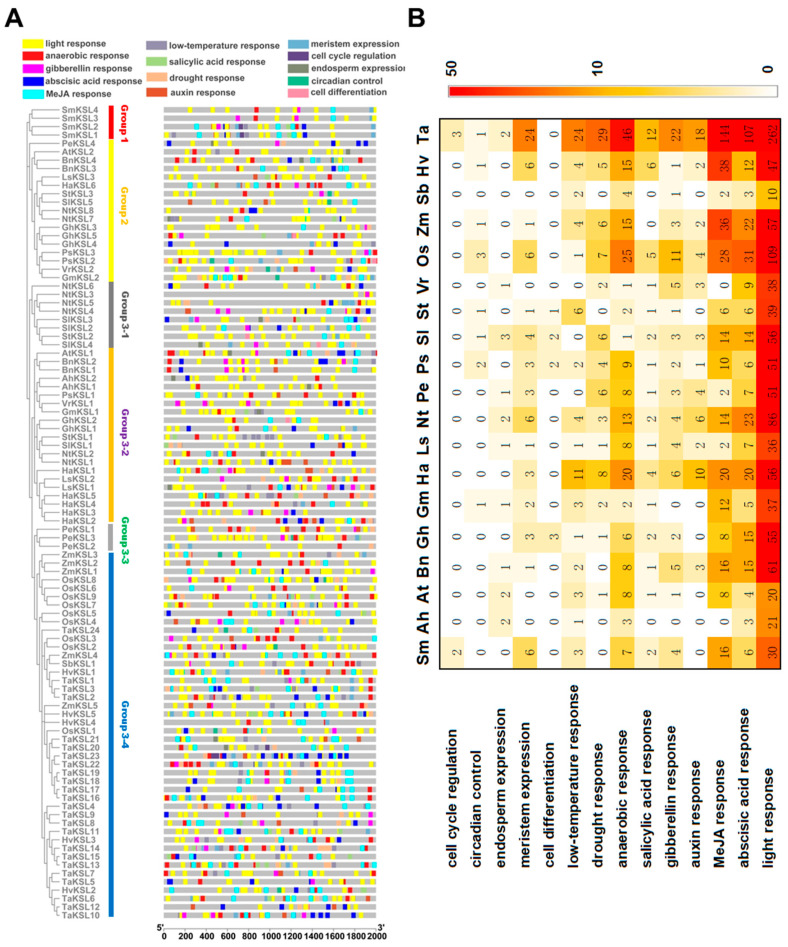
Promoter cis-element composition analysis of 97 *KSLs* promoters. (**A**) Distribution of cis-regulatory elements within the promoter regions of *KSL* gene family members. A 2000 bp region upstream from the transcription start site (displayed in gray) was examined for each *KSL* promoter. Colored bars represent different categories of regulatory elements. (**B**) Heatmap representation of the frequency of cis-regulatory elements in *KSL* gene promoters across 19 species. The color intensity corresponds to the abundance of specific cis-elements, with a gradient from lighter to darker red indicating increasing frequency.

Although various important metabolism products, including phytocassanes A-G and momilactones, which play significant roles in biotic stress like rice blast fungus and white leaf blight, can be formed by catalysis of paralogs of KSL, we were curious about the role of the *OsKSL* gene family in abiotic stress. Meanwhile, most of the functional *cis*-acting elements are concentrated within proximal promoters, usually spanning the region from −1000 bp to +200 bp relative to the transcription start site (TSS). Therefore, we analyzed the 2000 bp nucleotide sequence located upstream of the ATG initiation codon for all 97 *KSL* genes using the PlantCARE online tool. The types and their motif sequences of identified *cis*-acting elements are shown in the Supplementary Materials. The distribution and arrangement of 14 type elements within 97 *KSL* promoters are shown in Figure 3A. 

Figure 3B shows that the elements of light response were present in all 19 species, with the highest frequency among the 14 element types. This finding suggests that light response is a significant functional aspect associated with *KSL* genes. We also observed the presence of anaerobic response elements in all 19 plant species, as denoted by the red labels in Figure 3A. This widespread distribution emphasizes the importance and evolutionary conservation of *KSL* genes in anaerobic responses. Moreover, a relatively larger number of abscisic acid response elements (blue) and MeJA response elements (cyan) were observed across 19 plants. Although *KS* genes are vital in GA synthesis, it was noteworthy that gibberellin response elements were not detected in every *KSL* promoter. Furthermore, other *cis*-acting elements exhibited a less widespread distribution throughout the *KSL* genes of the 19 species. In conclusion, the *OsKSL* gene family possibly participates in light response, ABA response, and anaerobic response. As a result, the next experiments focused on these three abiotic stresses.

### 2.5. Collinearity Analysis of KSL Genes between Plant Species

**Figure 4:**
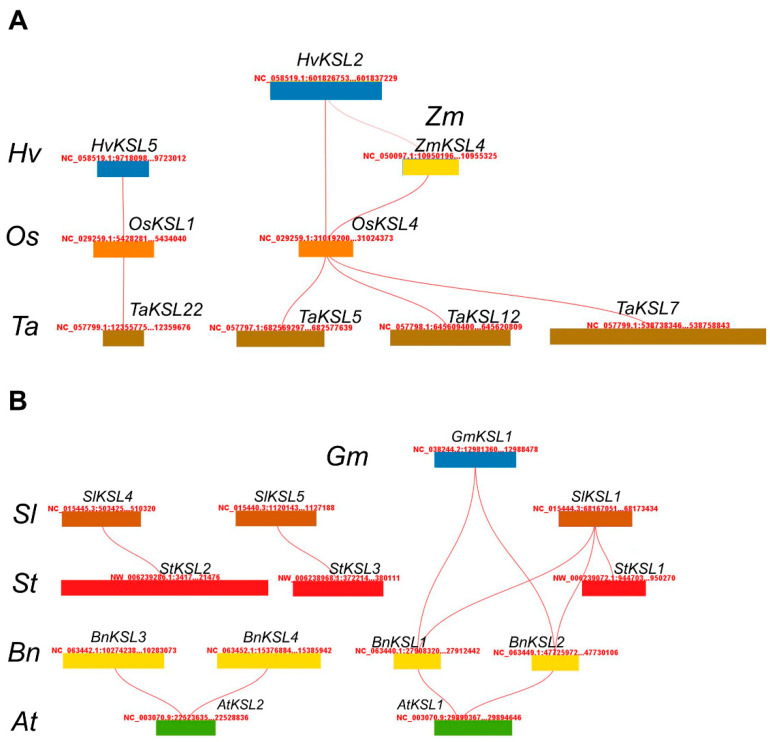
Comparative collinearity analysis of *KSL* genes across monocotyledonous and dicotyledonous species. (**A**) Monocotyledonous plant species illustrating synteny blocks containing *KSL* genes. The chromosomes of barley, rice, wheat, and maize are depicted, with lines connecting orthologous *KSL* gene regions. (**B**) Dicotyledonous plant species demonstrating conserved genomic segments with *KSL* genes. The representations include tomato, potato, oilseed rape, and thale cress with lines showing homologous gene correspondences.

To obtain insight into the evolutionary orthologous relationships of *KSL* genes among different plant species, a collinearity analysis of *KSL* genes was conducted (Figure S2). Then, based on the collinearity analysis between the two species, we made an overview diagram (Figure 4). Surprisingly, comparing all of the monocotyledon and dicotyledon pairs, we found that there was no direct *KSL* gene duplication across the two classes, implying that the gene function of the *KSL* family in monocots and dicots may be precisely regulated. Subsequently, a collinearity analysis of the *KSL* genes between monocots and dicots revealed good collinearity relationships between specific *KSL* genes (Figure 3 and Figure S2). For instance, among Poaceae species, including rice, wheat, maize, and barley, *OsKSL1* displayed collinearity with *HvKSL5* and *TaKSL22*, while *OsKSL4* showed collinearity with *HvKSL2*, *ZmKSL4*, and *TaKSL5*/*12*/*7*. This finding underscored the significance and high conservation of *OsKSL1* and *OsKSL4*. For dicots, more collinearity gene pairs were identified in the same family, such as Cruciferae (four gene pairs in *Arabidopsis* and *Brassica napus*) and Solanaceae (three gene pairs in tomato and potato). To confirm the results of the collinearity analysis, pairwise protein sequence alignments were conducted (Figures S3–S8), indicating the high homology of corresponding *KSL* pairs. 

We also made a collinear correlation of the *KSLs* gene in rice, but no collinear relationship was found, although the *OsKSL3*, *OsKSL7*, and *OsKSL9*, and *OsKSL4*, *OsKSL2* and *OsKSL5* genes formed two gene clusters in chromosome 2 and 4, respectively (Figure S9). This result suggested that the expansion of *OsKSL* family members most possibly depends on transposons rather than gene duplication. Notably, *OsKSL6* and *OsKSL8* were located in chromosomes 12 and 11, respectively.

### 2.6. Tissue Expression Patterns of KSL Genes in Rice

**Figure 5:**
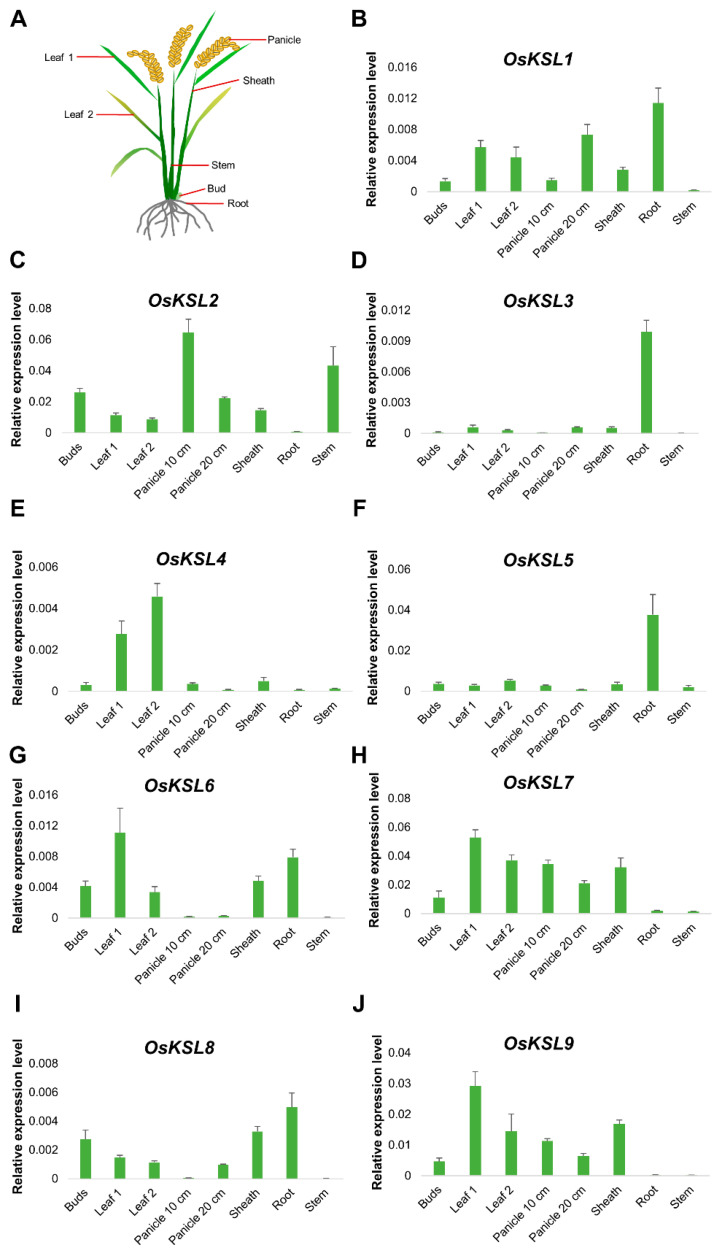
Tissue-specific expression of 9 *KSL* genes in rice. (**A**) Schematic illustration of the rice plant indicating the tissues sampled for gene expression analysis. (**B**–**J**) Relative expression analysis of 9 *OsKSL* genes in different tissues using qRT-PCR. RNA from various tissues (buds, leaf, panicle, sheath, root, and stem) of *Nip* (*n* = 3) was extracted. Three biological replications were performed. Bar graphs show means. Error bars represent ± SE (*n* = 3). *OsUBQ6* was used as internal reference (Table S2) [32].

Tissue-specific expression profiles are associated with the function of genes [29,30,31]. To investigate the characteristics of *OsKSL* expression in different tissues (bud, leaf, panicle, sheath, root, and stem), qRT-PCR analysis was performed. As shown in Figure 5, most of the *OsKSLs* were generally expressed in various tissues, with *OsKSL3* and *OsKSL5* preferentially expressed in the root and *OsKSL4* in the leaf. Interestingly, gene homology was not associated with tissue expression specificity, since *OsKSL2* had the highest homology with *OsKSL3* among the *OsKSL* gene family (Figure 1). 

### 2.7. Expression Patterns of OsKSL Genes under GA and PAC Treatment

**Figure 6:**
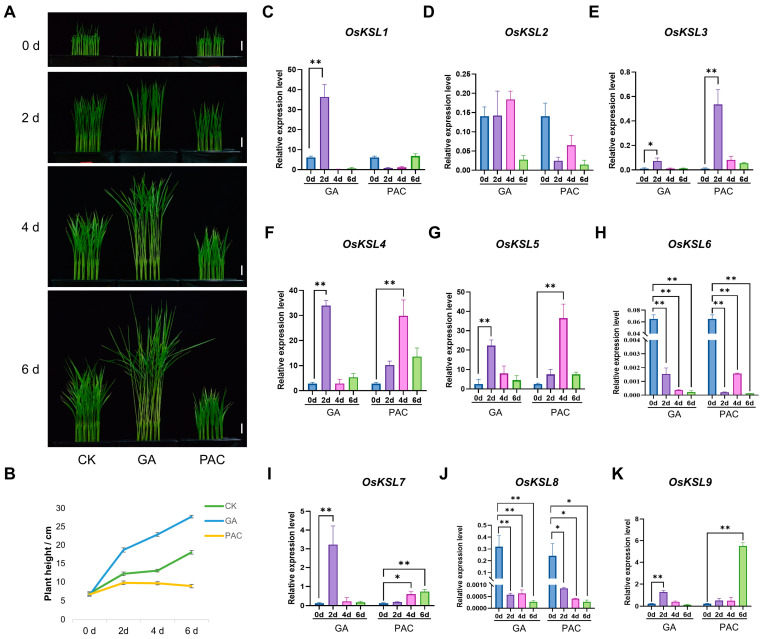
Expression analysis of *OsKSL* genes under GA and PAC treatment in rice. (**A**) Morphological comparison of 5-day-old seedlings of *Nipponbare* (*n* = 20) subjected to 10 μM GA or 10 μM PAC treatment at various time points (0, 2, 4, and 6 days). The left column represents Nip seedlings grown under standard conditions (CK). The central and right columns display Nip seedlings treated with 10 μM GA and 10 μM PAC, respectively. Scale bars = 1 cm. (**B**) Growth kinetics showing the elongation of Nip seedlings over time following GA and PAC treatments as described in panel A. *n* = 20. (**C**–**K**) Expression analysis of *KSL* genes under 10 μM GA or 10 μM PAC treatment in rice seedlings. Aerial tissues were sampled at designated time points (0, 2, 4, and 6 d) (*n* = 4) for RNA isolation and subsequent qRT-PCR. Significance levels are indicated as follows: * *p* < 0.05 and ** *p* < 0.01 (Student’s *t*-test). Error bars represent ± SE (*n* = 3). *OsUBQ6* was used as internal control (Table S2). Three biological replications were performed.

As the OsKSL2 protein can transfer *ent*-copalyl diphosphate (*ent*-CDP) into gibberellins, we wondered whether GA would influence *OsKSL* expression. Therefore, the 6-day seedling Nip (CK) was hydroponically cultured with 10 μM GA or 10 μM PAC, a GA synthesis inhibitor, for 6 days (Figure 6A). Nip supplemented with GA was higher than the control group, while those treated with PAC had the lowest height (Figure 6A,B). Next, qRT-PCR was used to detect the expressions of nine *OsKSL* genes under GA or PAC treatment (Figure 6C–K).

Unsurprisingly, the transcript level of *OsKSL2* was strongly repressed by PAC, although it did not seem to be affected by GA. The gene expression of *OsKSL1*, which encodes *syn*-pimara-7,15-diene synthase, was induced dramatically by GA, while there was an opposite trend in PAC treatment, suggesting it is possibly involved in the regulation of rice plant height [33]. Moreover, the expression patterns of the rest of the *OsKSLs* were consistent, regardless of GA or PAC treatment. For example, GA and PAC severely repressed *OsKSL6* and *OsKSL8* expressions, which implied that they are not associated with plant height or that every metabolic pathway is adjusted by distinct factors. In summary, *OsKSL1* and *OsKSL2* may play an important role in controlling plant height by participating in the GA pathway.

### 2.8. Responses of OsKSL Genes under Abiotic Stresses in Rice

Given the previous *cis*-element analysis results and the reports of KS related to pathogen infection, our study focused on the response of *KSL* genes to light, ABA, and anaerobic stress. Then, qRT-PCR analysis was used to verify whether these genes were responding to the above stresses. 

**Figure 7:**
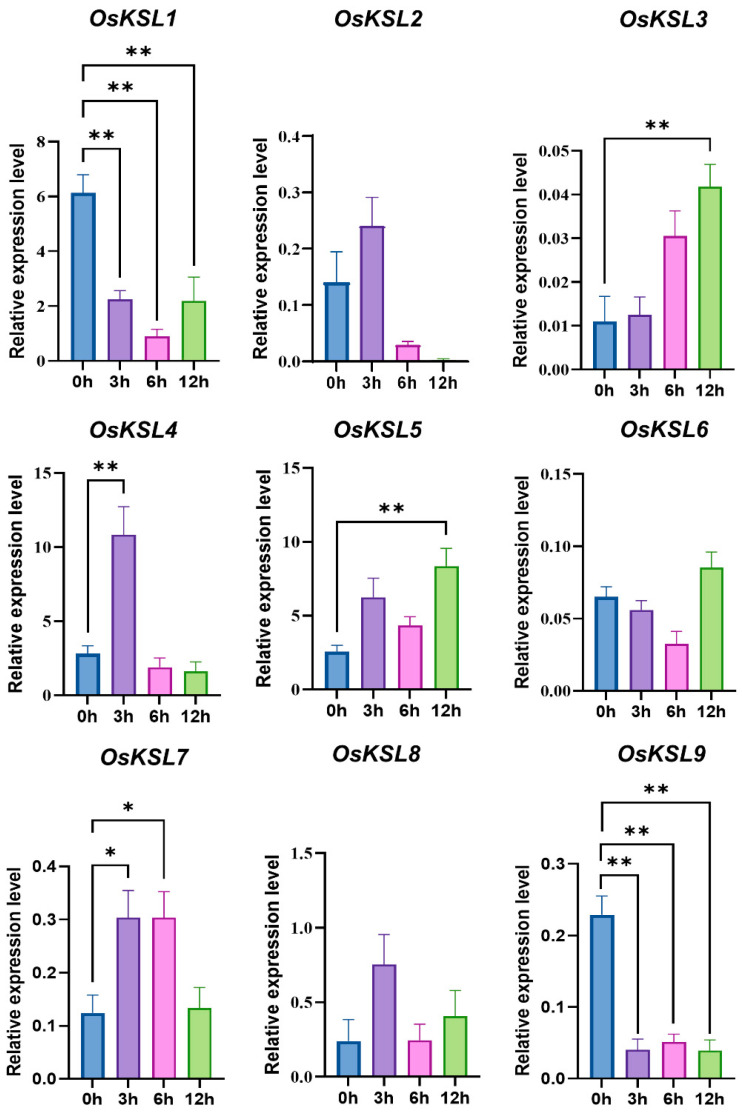
Expression profiles of *OsKSL* gene family in response to ABA treatment. Six-day-old seedlings were subjected to 50 μM ABA treatment. Aerial tissues were sampled at designated time points (0, 3, 6, and 12 h) (*n* = 4) for RNA isolation and subsequent qRT-PCR. * *p* < 0.05 and ** *p* < 0.01 (Student’s *t*-test). Error bars represent ± SE (*n* = 3). Three biological replications were performed. *OsUBQ6* was used as internal control.

**Figure 8:**
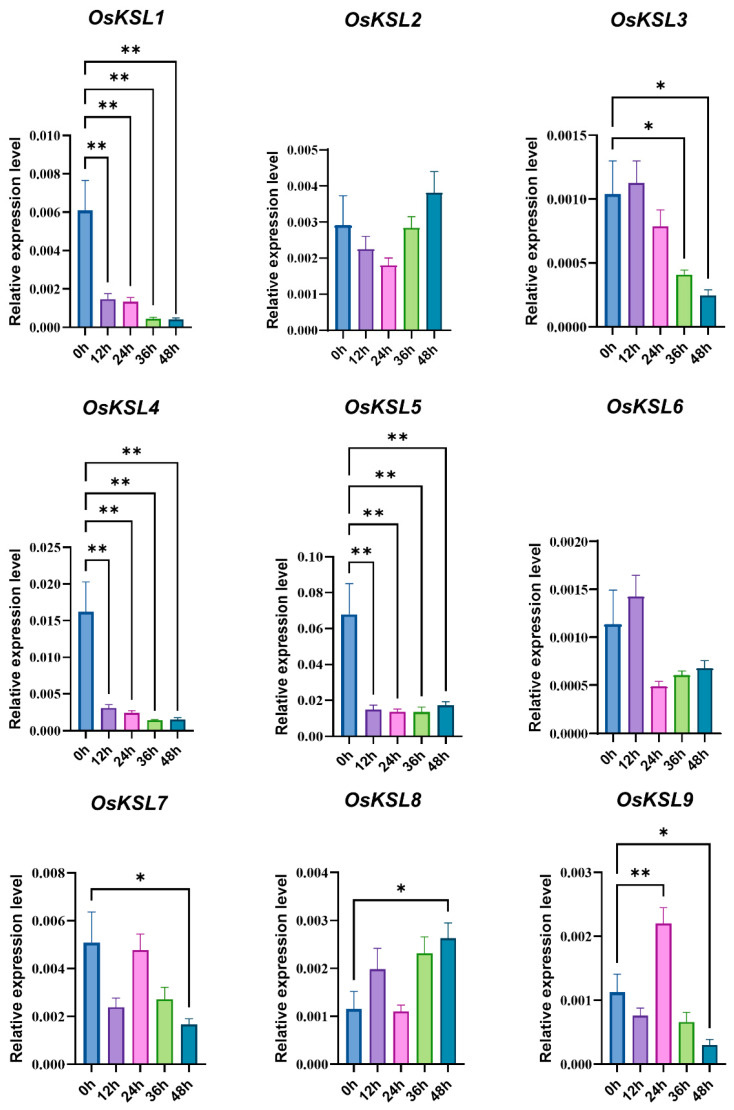
Expression profiles of *OsKSL* gene family in response to submergence stress. Six-day-old seedlings were immersed in 1/5 Hoagland solution. Aerial tissues were collected at designated time points (0, 12, 24, 36, and 48 h post-submergence) (*n* = 4) for RNA isolation and subsequent qRT-PCR analysis. * *p* < 0.05 and ** *p* < 0.01 (Student’s *t*-test). Three biological replications were performed. Error bars represent ± SE (*n* = 3). *OsUBQ6* was used as internal reference.

Surprisingly, only four genes (*OsKSL1*, *OsKSL3*, *OsKSL4*, and *OsKSL6*) responded to light with an inclined trend, while the light response element was the maximum element in the promoters of the *KSL* gene family of 19 species (Figure S10). The number of ABA response elements was followed by the figure for light. With ABA treatment, the expression profile of *OsKSLs* could be classified into three categories (Figure 7). Firstly, ABA could effectively suppress *OsKSL1* and *OsKSL9* expression. Secondly, the gene expression of *OsKSL3* gradually increased. Additionally, the expression for *OsKSL5* had a rising trend, although it fluctuated up and down. The last class members were *OsKSL4* and *OsKSL7*, which first grew and then fell. In terms of submergence, the expression of six *OsKSLs* except for *OsKSL2*, *OsKSL6*, and *OsKSL8* was repressed, although the figures for *OsKSL7* and *OsKSL9* after 24 h of treatment were higher than after 12 h treatment. Only *OsKSL8* was induced by anaerobic stress (Figure 8). In general, ABA and submergence impacted the majority of the *OsKSLs*’ expression, meaning that *OsKSLs* may work under these stresses.

## 3. Discussion

Previous research regarding *KSLs* focused on their biochemical functions [26,34] and evolutionary process [27,35], especially those that can form gene clusters with other genes involved in the same metabolic pathway, such as *OsKSL1* and *OsKSL3* [36], encoding *syn*-pimara-7,15-diene synthase and *ent*-cassa-12,15-diene synthase, respectively. Meanwhile, as the *KSL* family is responsible for the biosynthesis of phytohormone gibberellins and phytoalexins, which are associated with biotic stress, the functions of *KSLs* on biotic stress have been intensively documented in several studies. However, the role of the *KSL* family in rice remains uncertain, especially in abiotic stress. Therefore, we detected the *KSL* gene family in 19 species and analyzed their phylogenetic relationship, conserved motifs, and *cis*-acting elements of the promoter. Furthermore, the tissue expression profile and different responses of *OsKSL* family genes under hormone treatment (GA, PAC, and ABA), dark environment, and anaerobic stress were also explored. 

Based on their biochemical function, KSL family members can be divided into three categories: bifunctional cyclase, *ent-kaurene synthase*, and the enzymes associated with phytoalexin synthesis. Firstly, the bifunctional cyclases presented in fungi *Gibberella fujikuroi*, sporophytes *Physcomitrella patens*, and *Selaginella moellendorffii* were distinguishable from the monofunctional *ent-kaurene synthase* in higher plants [37]. In the KSL family of higher plants, *ent-kaurene synthase* (KS) was involved in GA synthesis to regulate plant growth and development, while *ent-kaurene synthase-like* (KSL) was involved in the synthesis of various phytoalexins to regulate plant defense response [34]. In rice, *OsKSL2* encodes an *ent-kaurene synthase* for GA biosynthesis, while *OsKSL1*, *OsKSL3*, *OsKSL6*, *OsKSL7*, *OsKSL8*, and *OsKSL9* encode *syn*-pimaradiene synthase, *ent*-cassadiene synthase, *ent*-sandaracopimaradiene synthase, *ent*-sandaracopimaradiene synthase, stemarene synthase, and *ent*-isokaurene synthase, respectively, for the biosynthesis of different kinds of phytoalexins, such as phytocassanes, momilactones, and oryzalexins [27]. 

Consistent with different biochemical functions, the phylogenetic tree of KSLs can be categorized into three clades (Figure 1). Firstly, group 1 contained bifunctional enzymes of sporophyte plants. Secondly, group 2 contained KSL members involved in the synthesis of phytoalexins in dicotyledonous plants. For example, in group 2, *AtKSL2*, *BnKSL3*, *BnKSL4*, *NtKSL7*, *NtKSL8*, *SlKSL5*, *StKSL3*, *GhKSL3*, *GhKSL4*, *GhKSL5*, and *VrKSL2* encode geranyl linalool synthase for phytoalexins synthesis instead of *ent*-kaurene synthesis. *LsKSL3*, *HaKSL6*, *PsKSL2*, *PsKSL3*, and *PeKSL4* all encode S-linalool synthase instead of KS, which should also be related to phytoalexin synthesis. As expected, in group 3-2, *AtKSL1*, *BnKSL1*, *BnKSL2*, *SlKSL1*, *StKSL1*, and *GmKSL1* encode KS, which should be involved in GA synthesis. In addition, in group 3-3, *PeKSL1*, *PeKSL2*, and *PeKSL3* encode KS as well, which indicates that group 3-1, group 3-2, and group 3-3 were the groups of KS with differences. Thirdly, all KSLs in monocotyledons were grouped into group 3-4, indicating that the protein sequences of KSLs in monocots and dicots were obviously distinguishable. The classification of KSLs in monocotyledons was similar to that in dicotyledons. For example, *OsKSL2*, *ZmKSL4*, and *SbKSL1* encoding KS were grouped. *OsKSL1*, *TaKSL20*, *TaKSL21*, *TaKSL22*, and so on, which encode pimaradiene synthase, were classified together, as were *OsKSL6*, *OsKSL7*, *OsKSL8*, and *OsKSL9*, which participate in phytoalexin synthesis. 

To obtain insight into the evolutionary orthologous relationships of *KSL* genes among different plant species, a collinearity analysis of *KSL* genes was conducted (Figure 4). The species with a closed evolutionary relationship had more collinearity pairs, such as tomato and potato. Generally, the homology genes of different species have similar functions [38]. Thus, we can speculate the function of an unknown gene according to the functional study of its homology gene. Compared to WT, the *gmksl4*/*ks3-1* maize mutant reduces the production of endogenous gibberellin, but shows a stronger drought resistance, suggesting that the homology genes of *GmKSL4* (*HvKSL2* and *OsKSL4*) may alter plants’ drought resistance [7]. In Arabidopsis, *AtKSL1*/*AtKS* overexpression lines show similar phenotypes to WT for flowering time and rosette development [39]. It is possible that *BnKSL1* and *BnKSL2*, the homology genes of *AtKSL1*, cannot influence plant PAC tolerance. Surprisingly, we found that there was no *KSL* gene duplication shared between monocotyledons and dicotyledons. As the differentiation degree between the monocots and dicots is extremely high, the evolutionary track of *KSLs* between them is subtle. Selecting the evolutionary node species and performing collinearity may be helpful. For instance, *GmKSL1*, *SlKSL1*, and *AtKSL1* cannot form collinearity pairs. It was *BnKSL1* and *BnKSL2* that tied them together. 

To investigate the expression profile of *OsKSLs* in different tissues, qRT-PCR analysis was performed. It was found that the homology between *OsKSL2* and *OsKSL3* was high; however, their expression patterns were different. Firstly, their promoters were analyzed and there were large differences in the two promoter sequences, which may be the main reason why the expression patterns of these two genes exhibit differences. Additionally, we also analyzed their gene structures. *OsKSL3* has a long intron in the 5′ terminal while *OsKSL2* does not, implying that the gene structure might influence the expression level.

To explore the role of *OsKSLs* in abiotic stress, the *cis*-acting elements of *KSL* promoters in 19 species were detected and accounted (Figure 3). The number of components in order from most to least is light response, MeJA, ABA response, and anaerobic response. Although the light response element appeared most frequently, only four genes responded to darkness. The transcript levels of *OsKSL1*, *OsKSL3*, and *OsKSL7* represented three patterns of ABA response, indicating that these genes can be used to explore their role in the ABA pathway. Interestingly, submergence influenced seven *OsKSLs*’ gene expression. Based on tissue expression and expression profiles in diverse situations, *OsKSL1*, *OsKSL3*, and *OsKSL4* were optimal genes for follow-up experiments, such as observing physiological phenotypes of mutants or overexpression lines under the above treatments and investigating the underlying regulatory mechanism [40,41].

We also found that no matter the species or gene families, these four types of *cis*-acting elements have the most frequent occurrence [42,43]. It is possible that plants are required to be sensitive to these stimuli for survival or that the algorithm for analyzing promoters needs to be improved. Also, the response of *KSLs* to other environmental stimuli (e.g., UV and CuCl_2_) should be assessed. Finally, this study systematically analyzed the KSL family of 19 species and preliminarily studied the role of *OsKSLs* in several hormones and abiotic stresses that provide the theoretical basis for reverse genetics. Further functional verification should be carried out to understand the role of *OsKSL* family genes under different conditions.

## 4. Materials and Methods

### 4.1. Identification of KSL Genes among 19 Species

Considering the role of evolutionary processes and economics, we selected 19 plant species. To perform genome-wide identification of the *KSL* gene family across these species, we downloaded 19 species genomes and their corresponding annotation documents from the NCBI database (https://www.ncbi.nlm.nih.gov/, accessed on 18 April 2023). Meanwhile, the Hidden Markov models (HMMs) of the Terpene_ synth domain (Pfam: PF01397) and Terpene_synth_C domain (Pfam: PF03936) were downloaded from the Pfam database (http://www.sanger.ac.uk/Software/Pfam, accessed on 18 April 2023). The potential *KSL* genes were identified using TBtools, with statistical significance indicated by an e-value threshold of <10^−5^ [44]. These retrieved sequences were screened by the SMART database (http://smart.embl-heidelberg.de/, accessed on 18 April 2023). After removing redundant transcripts, the dataset containing 97 KS proteins of 19 species was used for further analysis. The prediction of physicochemical properties, like the isoelectric point and molecular weight, was calculated by TBtools (v2.069).

### 4.2. Phylogenetic and Protein Structure Analyses of the KSL Family

The above verified KSL protein sequences were subjected to multiple sequence alignments using MUSCLE alignment. The neighbor-joining tree was constructed by MEGA 7 with a bootstrap test (1000 replicates) [44]. Finally, the Interactive Tree of Life (iTOL) web (https://itol.embl.de/, accessed on 18 April 2023) refined and visualized the phylogenetic tree.

The conserved motif and domain analyses of KSL proteins were elucidated by using the online MEME website (https://meme-suite.org/meme/tools/meme, accessed on 18 April 2023) and Batch CD-Search (https://www.ncbi.nlm.nih.gov/Structure/bwrpsb/bwrpsb.cgi, accessed on 18 April 2023), respectively [45,46]. Both of these results were combined by TBtools [47].

### 4.3. Collinearity Analysis and Chromosomal Distribution of KSL Genes

To uncover the evolutional process of the *KSL* family among dicots and monocots, collinearity analysis was performed by Multiple Collinearity Scan (MCScanX), a toolkit from TBtools. Species genomes and their corresponding annotation documents were required to complete this analysis. To confirm the results of the collinearity analysis, multiple sequence alignments were performed by the online website ESPript (https://espript.ibcp.fr, accessed on 18 April 2023) [48]. Also, TBtools was responsive enough to calculate and visual *KSLs* loci on chromosomes. 

### 4.4. Cis-Acting Element Analysis of KSL Promoters

The 2000 bp upstream of *KSLs* was recognized as their potential promoter. Here, 97 *KSLs* promoter sequences were retrieved by the Gtf/Gff3 Sequences Extract toolkit. Then, we used PlantCARE (https://bioinformatics.psb.ugent.be/webtools/plantcare/html/, accessed on 18 April 2023) to calculate all known *cis*-acting elements. The xls. document produced by PlantCARE was required to be modified, such as removing redundant and uncharacterized *cis*-acting elements. The resultant document was used to visualize the results by TBtools.

### 4.5. Plant Materials and Stress Treatments

In this study, *Nipponbare* (Nip) was used as the experimental material to analyze the expression profile of *OsKSL* family members. The seeds were placed on filter papers and soaked in deionized water at 30 °C for 2 days. After germination, the seeds grew in hydroponic boxes with 1/5 Hoagland Nutrient Solution (PHYGENE) at 32 °C (light) and 25 °C (darkness) for a week. The hydroponic experiment was performed with a 14 h light and 10 h darkness cycle. Six-day-old seedlings were used for a variety of hormone and abiotic stress treatments. Configured hormones (10 μM GA, 10 μM PAC, and 50 μM ABA) were added directly into the nutrient solution. The seedlings grew in darkness and were submerged for the light experiment and anaerobic treatment, respectively. Every experiment was performed with triple biological repeats and the representative results were shown. The aerial tissues of 4 units were sampled and the photographs of GA and PAC were taken at the described time point for RT-qPCR analysis. Triple biological replications were used in the RT-qPCR assay.

### 4.6. RNA Extraction and Quantitative/Real-Time-PCR (RT-qPCR) Analysis

The total RNA of the abovementioned plant materials was extracted by TRIzol (Coolaber) and 1.2 μg total RNA was reverse-transcribed into cDNA (Vazyme, Nanjing, China) for subsequent experiments. The qRT-PCR experiment was performed using the CFX384 real-time system (Biorad, America, CA, USA). The ChamQ universal SYBR qPCR Master Mix (Vazyme, China) reagent was utilized. Every experiment had three biological duplications. The reference gene was the *OsUBQ* of *Oryza sativa*. The primers used in the experiment are listed in Table S2.

## 5. Conclusions

We identified the KSL family of 19 species by domain analysis and grouped 97 KSL family proteins into three categories. A collinearity analysis of *KSL* among Poaceae indicated that the *KSL* gene may independently evolve and *OsKSL1* and *OsKSL4* likely play a significant role in the evolutionary process. Tissue expression analysis showed that only *OsKSL3* and *OsKSL5* were specifically expressed in the root and *OsKSL4* in the leaf. The qRT-PCR results suggested that *OsKSL1* was extremely likely to be involved in the regulation of plant height, and *OsKSL1*, *OsKSL3*, and *OsKSL4* can be candidate genes for abiotic stress.

## Figures and Tables

**Table 1:** The characteristics of 97 KSL family proteins.

Species	Protein ID	Rename	Number of Amino Acids	MW (Da)	PI
*Selaginella moellendorffii*	XP_024539099.1	SmKSL1	754	86,571.79	6.45
	XP_024525392.1	SmKSL2	772	88,726.37	6.49
	XP_024539123.1	SmKSL3	752	86,068.1	6.23
	XP_024525512.1	SmKSL4	758	86,811.87	6.06
*Arabidopsis* *thaliana*	NP_178064.1	AtKSL1	785	89,622.17	5.65
	NP_564772.1	AtKSL2	877	101,895.31	6.12
*Brassica* *napus*	XP_048592391.1	BnKSL1	779	89,030.61	5.25
	XP_022560479.1	BnKSL2	781	89,085.62	5.4
	XP_013718286.1	BnKSL3	872	101,213.18	5.71
	XP_013690843.2	BnKSL4	870	101,147.98	5.79
*Nicotiana* *tabacum*	XP_016473917.1	NtKSL1	841	94,993.35	5.98
	XP_016507998.1	NtKSL2	847	95,694.9	5.66
	XP_016497928.1	NtKSL3	747	86,923.71	5.65
	XP_016485246.1	NtKSL4	797	91,793.03	5.58
	XP_016506641.1	NtKSL5	797	91,831.18	5.78
	XP_016453352.1	NtKSL6	753	87,080.29	5.49
	XP_016495633.1	NtKSL7	863	98,863.6	6.02
	XP_016493341.1	NtKSL8	865	98,910.15	6.43
*Glycine* *max*	XP_006585394.1	GmKSL1	834	95,150.3	6.16
	XP_040864136.1	GmKSL2	836	96,446.96	6.3
*Phalaenopsis equestris*	XP_020579527.1	PeKSL1	799	90,548.33	5.74
	XP_020599757.1	PeKSL2	841	95,840.83	5.93
	XP_020588364.1	PeKSL3	896	101,568.45	6.25
	XP_020576697.1	PeKSL4	845	97,840.56	6.26
*Helianthus* *annuus*	XP_022027301.1	HaKSL1	786	90,239.11	5.8
	XP_035840816.1	HaKSL2	774	88,384.53	5.15
	XP_035840815.1	HaKSL3	772	88,103.09	5.22
	XP_022018205.1	HaKSL4	803	91,637.34	5.32
	XP_022017950.1	HaKSL5	774	88,098.23	5.03
	XP_022042326.1	HaKSL6	831	96,102.28	5.61
*Lactuca* *sativa*	XP_042756891.1	LsKSL1	818	92,809.35	5.62
	XP_023729144.1	LsKSL2	794	90,505.71	5.75
	XP_023752639.1	LsKSL3	843	97,936.55	5.74
*Oryza* *sativa*	XP_015633583.1	OsKSL1	842	94,956.28	5.2
	XP_015633664.1	OsKSL2	813	92,036.64	6.1
	XP_025878382.1	OsKSL3	819	90,986.5	5.1
	XP_025880472.1	OsKSL4	763	86,820.23	5.18
	XP_015634420.1	OsKSL5	819	91,265.02	5.82
	XP_015618915.1	OsKSL6	815	91,929.82	5.63
	XP_015625948.1	OsKSL7	821	92,376.71	5.48
	XP_015617512.1	OsKSL8	822	90,258.11	5.41
	XP_015625944.1	OsKSL9	836	94,130.9	5.57
*Zea* *mays*	NP_001169726.1	ZmKSL1	848	95,107.22	5.21
	XP_023158035.1	ZmKSL2	828	92,497.13	6.38
	NP_001146027.1	ZmKSL3	800	89,179.11	5.3
	NP_001348116.1	ZmKSL4	802	90,809.89	6
	NP_001348122.1	ZmKSL5	840	94,759.96	5.51
*Arachis* *hypogaea*	XP_025610006.1	AhKSL1	799	91,257.36	5.68
	XP_025672039.1	AhKSL2	799	91,234.32	5.72
*Hordeum* *vulgare*	XP_044967658.1	HvKSL1	850	95,794.79	6.04
	XP_044967650.1	HvKSL2	752	85,663.93	5.83
	XP_044968830.1	HvKSL3	779	88,905.02	5.31
	XP_044947960.1	HvKSL4	848	96,556.59	6.6
	XP_044969121.1	HvKSL5	835	94,720.6	5.58
*Pisum* *sativum*	XP_050917776.1	PsKSL1	798	90,970.91	5.92
	XP_050902783.1	PsKSL2	811	93,694.93	7.29
	XP_050903277.1	PsKSL3	805	93,346.48	6.62
*Solanum lycopersicum*	NP_001307929.1	SlKSL1	820	92,593.64	6.02
	NP_001234629.1	SlKSL2	778	90,819.41	6.62
	XP_010324501.1	SlKSL3	771	90,420.53	6.24
	XP_025888303.1	SlKSL4	726	85,054.02	6.43
	NP_001289840.1	SlKSL5	821	95,168.79	6.55
*Solanum tuberosum*	XP_006346019.1	StKSL1	826	93,374.76	6.57
	XP_015170847.1	StKSL2	779	91,009.28	5.82
	XP_015162412.1	StKSL3	829	95,979.35	6.45
*Sorghum* *bicolor*	XP_021319614.1	SbKSL1	808	91,045.28	5.8
*Vigna* *radiata*	XP_014499923.1	VrKSL1	786	89,825.19	5.84
	XP_022639296.1	VrKSL2	836	95,775.88	5.96
*Gossypium* *hirsutum*	NP_001314116.1	GhKSL1	780	89,076.64	5.9
	XP_016701676.2	GhKSL2	780	88,961.54	6.07
	XP_016698390.2	GhKSL3	771	88,182.81	5.13
	XP_040949849.1	GhKSL4	847	96,284.19	5.35
	XP_016698869.1	GhKSL5	849	96,891.9	5.39
*Triticum* *aestivum*	XP_044325790.1	TaKSL1	846	95,122.97	6.4
	XP_044333990.1	TaKSL2	852	95,793.99	6.62
	XP_044458743.1	TaKSL3	852	95,841.85	6.43
	XP_044460415.1	TaKSL4	950	105,991.71	5.97
	XP_044460359.1	TaKSL5	814	91,810.02	5.39
	XP_044335649.1	TaKSL6	771	87,797.28	5.45
	XP_044335648.1	TaKSL7	865	97,853.75	5.18
	XP_044329066.1	TaKSL8	862	97,315.15	6.01
	XP_044318592.1	TaKSL9	868	98,416.47	6.21
	XP_044320364.1	TaKSL10	857	96,689.59	6.4
	XP_044325785.1	TaKSL11	856	97,289.12	5.92
	XP_044318496.1	TaKSL12	908	101,745.39	5.61
	XP_044335652.1	TaKSL13	776	88,291.32	5.51
	XP_044460363.1	TaKSL14	776	88,189.19	5.66
	XP_044318500.1	TaKSL15	765	87,063.86	5.13
	XP_044327332.1	TaKSL16	837	95,184.83	6.2
	XP_044452386.1	TaKSL17	837	95,160.73	6.02
	XP_044318934.1	TaKSL18	835	95,125.68	5.84
	XP_044318936.1	TaKSL19	835	95,139.71	5.84
	XP_044327331.1	TaKSL20	837	94,852.07	5.4
	XP_044452380.1	TaKSL21	837	94,588.97	5.6
	XP_044335422.1	TaKSL22	839	94,704.17	5.53
	XP_044452378.1	TaKSL23	839	94,842.34	5.77
	XP_044408678.1	TaKSL24	716	81,002.53	5.12

**Table 2:** Distribution and subfamily classification of KSL proteins within the 19 plant species.

Species	Total	Group 1	Group 2	Group 3			
				3-1	3-2	3-3	3-4
Sm	4	4	-	-	-	-	-
At	2	-	1	-	1	-	-
Bn	4	-	2	-	2	-	-
Pe	4	-	1	-	-	3	-
Nt	8	-	2	4	2	-	-
Sl	5	-	1	3	1	-	-
St	3	-	1	1	1	-	-
Ls	3	-	1	-	2	-	-
Ha	6	-	1	-	5	-	-
Gm	2	-	1	-	1	-	-
Ps	3	-	2	-	1	-	-
Vr	2	-	1	-	1	-	-
Ah	2	-	-	-	2	-	-
Gh	5	-	3	-	2	-	-
Os	9	-	-	-	-	-	9
Zm	5	-	-	-	-	-	5
Ta	21	-	-	-	-	-	21
Hv	5	-	-	-	-	-	5
Sb	1	-	-	-	-	-	1

## Data Availability

Data is contained within the article and Supplementary Material.

## Supplementary Materials

The following supporting information can be downloaded at: https://www.mdpi.com/article/10.3390/ijms25105513/s1.

## References

[1] Yamaguchi S., Sun T., Kawaide H., Kamiya Y. (1998). The GA2 locus of Arabidopsis thaliana encodes *ent-kaurene synthase* of gibberellin biosynthesis. Plant Physiol..

[2] Wang Y., Zhao J., Lu W., Deng D. (2017). Gibberellin in plant height control: Old player, new story. Plant Cell Rep..

[3] Hedden P. (2020). The Current Status of Research on Gibberellin Biosynthesis. Plant Cell Physiol..

[4] Silverstone A.L., Chang C., Krol E., Sun T.P. (1997). Developmental regulation of the gibberellin biosynthetic gene GA1 in Arabidopsis thaliana. Plant J..

[5] Tezuka D., Ito A., Mitsuhashi W., Toyomasu T., Imai R. (2015). The rice *ent-kaurene synthase like* 2 encodes a functional ent-beyerene synthase. Biochem. Biophys. Res. Commun..

[6] Helliwell C.A., Sullivan J.A., Mould R.M., Gray J.C., Peacock W.J., Dennis E.S. (2001). A plastid envelope location of Arabidopsis ent-kaurene oxidase links the plastid and endoplasmic reticulum steps of the gibberellin biosynthesis pathway. Plant J..

[7] Wu H., Bai B., Lu X., Li H. (2023). A gibberellin-deficient maize mutant exhibits altered plant height, stem strength and drought tolerance. Plant Cell Rep..

[8] Gao Y., Huang S., Qu G., Fu W., Zhang M., Liu Z., Feng H. (2020). The mutation of *ent-kaurene synthase*, a key enzyme involved in gibberellin biosynthesis, confers a non-heading phenotype to Chinese cabbage (*Brassica rapa* L. ssp. pekinensis). Hortic. Res..

[9] Li Z.F., Guo Y., Ou L., Hong H., Wang J., Liu Z.X., Guo B., Zhang L., Qiu L. (2018). Identification of the dwarf gene GmDW1 in soybean (*Glycine max* L.) by combining mapping-by-sequencing and linkage analysis. Theor. Appl. Genet..

[10] Miyazaki S., Nakajima M., Kawaide H. (2015). Hormonal diterpenoids derived from ent-kaurenoic acid are involved in the blue-light avoidance response of Physcomitrella patens. Plant Signal. Behav..

[11] Hayashi K., Horie K., Hiwatashi Y., Kawaide H., Yamaguchi S., Hanada A., Nakashima T., Nakajima M., Mander L.N., Yamane H. (2010). Endogenous diterpenes derived from ent-kaurene, a common gibberellin precursor, regulate protonema differentiation of the moss Physcomitrella patens. Plant Physiol..

[12] Shimane M., Ueno Y., Morisaki K., Oogami S., Natsume M., Hayashi K., Nozaki H., Kawaide H. (2014). Molecular evolution of the substrate specificity of *ent-kaurene synthases* to adapt to gibberellin biosynthesis in land plants. Biochem. J..

[13] Zhou K., Xu M., Tiernan M., Xie Q., Toyomasu T., Sugawara C., Oku M., Usui M., Mitsuhashi W., Chono M. (2012). Functional characterization of wheat ent-kaurene(-like) synthases indicates continuing evolution of labdane-related diterpenoid metabolism in the cereals. Phytochemistry.

[14] Itoh A., Nakazato S., Wakabayashi H., Hamano A., Shenton M.R., Miyamoto K., Mitsuhashi W., Okada K., Toyomasu T. (2021). Functional kaurene-synthase-like diterpene synthases lacking a gamma domain are widely present in Oryza and related species. Biosci. Biotechnol. Biochem..

[15] Toyomasu T., Shenton M.R., Okada K. (2020). Evolution of Labdane-Related Diterpene Synthases in Cereals. Plant Cell Physiol..

[16] Shimada T., Minato S., Hasegawa Y., Miyamoto K., Minato Y., Shenton M.R., Okada K., Kawaide H., Toyomasu T. (2023). Characterization of diterpene synthase genes in Brachypodium distachyon, a monocotyledonous model plant, provides evolutionary insight into their multiple homologs in cereals. Biosci. Biotechnol. Biochem..

[17] Toyomasu T., Kawaide H., Ishizaki A., Shinoda S., Otsuka M., Mitsuhashi W., Sassa T. (2000). Cloning of a full-length cDNA encoding *ent-kaurene synthase* from Gibberella fujikuroi: Functional analysis of a bifunctional diterpene cyclase. Biosci. Biotechnol. Biochem..

[18] Kawaide H., Imai R., Sassa T., Kamiya Y. (1997). *ent-kaurene synthase* from the fungus Phaeosphaeria sp. L487. cDNA isolation, characterization, and bacterial expression of a bifunctional diterpene cyclase in fungal gibberellin biosynthesis. J. Biol. Chem..

[19] Hayashi K., Kawaide H., Notomi M., Sakigi Y., Matsuo A., Nozaki H. (2006). Identification and functional analysis of bifunctional *ent-kaurene synthase* from the moss Physcomitrella patens. FEBS Lett..

[20] Anterola A., Shanle E., Mansouri K., Schuette S., Renzaglia K. (2009). Gibberellin precursor is involved in spore germination in the moss Physcomitrella patens. Planta.

[21] Prisic S., Xu J., Coates R.M., Peters R.J. (2007). Probing the role of the DXDD motif in Class II diterpene cyclases. Chembiochem.

[22] Christianson D.W. (2006). Structural biology and chemistry of the terpenoid cyclases. Chem. Rev..

[23] Yamaguchi S. (2008). Gibberellin metabolism and its regulation. Annu. Rev. Plant Biol..

[24] Cho M.H., Lee S.W. (2015). Phenolic Phytoalexins in Rice: Biological Functions and Biosynthesis. Int. J. Mol. Sci..

[25] Kariya K., Ube N., Ueno M., Teraishi M., Okumoto Y., Mori N., Ueno K., Ishihara A. (2020). Natural variation of diterpenoid phytoalexins in cultivated and wild rice species. Phytochemistry.

[26] Xu M., Wilderman P.R., Morrone D., Xu J., Roy A., Margis-Pinheiro M., Upadhyaya N.M., Coates R.M., Peters R.J. (2007). Functional characterization of the rice kaurene synthase-like gene family. Phytochemistry.

[27] Toyomasu T., Miyamoto K., Shenton M.R., Sakai A., Sugawara C., Horie K., Kawaide H., Hasegawa M., Chuba M., Mitsuhashi W. (2016). Characterization and evolutionary analysis of *ent-kaurene synthase like* genes from the wild rice species Oryza rufipogon. Biochem. Biophys. Res. Commun..

[28] Fu J., Ren F., Lu X., Mao H., Xu M., Degenhardt J., Peters R.J., Wang Q. (2016). A Tandem Array of *ent-kaurene synthases* in Maize with Roles in Gibberellin and More Specialized Metabolism. Plant Physiol..

[29] Zhang M., Cao Y., Wang Z., Wang Z.-q., Shi J., Liang X., Song W., Chen Q., Lai J., Jiang C. (2018). A retrotransposon in an HKT1 family sodium transporter causes variation of leaf Na+ exclusion and salt tolerance in maize. New Phytologist..

[30] Zhang M., Liang X., Wang L., Cao Y., Song W., Shi J., Lai J., Jiang C. (2019). A HAK family Na(+) transporter confers natural variation of salt tolerance in maize. Nat. Plants.

[31] Frisse A., Pimenta M.J., Lange T. (2003). Expression studies of gibberellin oxidases in developing pumpkin seeds. Plant Physiol..

[32] Cai M., Zhu S., Wu M., Zheng X., Wang J., Zhou L., Zheng T., Cui S., Zhou S., Li C. (2021). DHD4, a CONSTANS-like family transcription factor, delays heading date by affecting the formation of the FAC complex in rice. Mol. Plant.

[33] Teng Y., Cai M., Xie Q., Liu Q., Zhang H., Chen T. (2023). BEAR1, a bHLH transcription factor, controls seedling growth by regulating gibberellins biosynthesis in rice. Crop J..

[34] Margis-Pinheiro M., Zhou X.R., Zhu Q.H., Dennis E.S., Upadhyaya N.M. (2005). Isolation and characterization of a Ds-tagged rice (*Oryza sativa* L.) GA-responsive dwarf mutant defective in an early step of the gibberellin biosynthesis pathway. Plant Cell Rep..

[35] Miyamoto K., Fujita M., Shenton M.R., Akashi S., Sugawara C., Sakai A., Horie K., Hasegawa M., Kawaide H., Mitsuhashi W. (2016). Evolutionary trajectory of phytoalexin biosynthetic gene clusters in rice. Plant J..

[36] Kitaoka N., Zhang J., Oyagbenro R.K., Brown B., Wu Y., Yang B., Li Z., Peters R.J. (2021). Interdependent evolution of biosynthetic gene clusters for momilactone production in rice. Plant Cell.

[37] Sakamoto T., Miura K., Itoh H., Tatsumi T., Ueguchi-Tanaka M., Ishiyama K., Kobayashi M., Agrawal G.K., Takeda S., Abe K. (2004). An overview of gibberellin metabolism enzyme genes and their related mutants in rice. Plant Physiol..

[38] Chen W., Chen L., Zhang X., Yang N., Guo J., Wang M., Ji S., Zhao X., Yin P., Cai L. (2022). Convergent selection of a WD40 protein that enhances grain yield in maize and rice. Science.

[39] Fleet C.M., Yamaguchi S., Hanada A., Kawaide H., David C.J., Kamiya Y., Sun T.-p. (2003). Overexpression of AtCPS and AtKS in Arabidopsis Confers Increased ent-Kaurene Production But No Increase in Bioactive Gibberellins. Plant Physiol..

[40] Okada A., Okada K., Miyamoto K., Koga J., Shibuya N., Nojiri H., Yamane H. (2009). OsTGAP1, a bZIP transcription factor, coordinately regulates the inductive production of diterpenoid phytoalexins in rice. J. Biol. Chem..

[41] Wang L., Fu J., Shen Q., Wang Q. (2023). OsWRKY10 extensively activates multiple rice diterpenoid phytoalexin biosynthesis genes to enhance rice blast resistance. Plant J..

[42] Sun M., Cai M., Zeng Q., Han Y., Zhang S., Wang Y., Xie Q., Chen Y., Zeng Y., Chen T. (2023). Genome-Wide Identification and Expression Analysis of UBiA Family Genes Associated with Abiotic Stress in Sunflowers (*Helianthus annuus* L.). Int. J. Mol. Sci..

[43] Han Y., Cai M., Zhang S., Chai J., Sun M., Wang Y., Xie Q., Chen Y., Wang H., Chen T. (2022). Genome-Wide Identification of AP2/ERF Transcription Factor Family and Functional Analysis of DcAP2/ERF#96 Associated with Abiotic Stress in Dendrobium catenatum. Int. J. Mol. Sci..

[44] Chen C., Chen H., Zhang Y., Thomas H.R., Frank M.H., He Y., Xia R. (2020). TBtools: An Integrative Toolkit Developed for Interactive Analyses of Big Biological Data. Mol. Plant.

[45] Kumar S., Stecher G., Tamura K. (2016). MEGA7: Molecular Evolutionary Genetics Analysis Version 7.0 for Bigger Datasets. Mol. Biol. Evol..

[46] Wang J., Chitsaz F., Derbyshire M.K., Gonzales N.R., Gwadz M., Lu S., Marchler G.H., Song J.S., Thanki N., Yamashita R.A. (2023). The conserved domain database in 2023. Nucleic Acids Res..

[47] Bailey T.L., Johnson J., Grant C.E., Noble W.S. (2015). The MEME Suite. Nucleic Acids Res..

[48] Robert X., Gouet P. (2014). Deciphering key features in protein structures with the new ENDscript server. Nucleic Acids Res..

