# Supplementary material for: Genome-Wide Identification and Expression Analysis of ent-kaurene synthase-like Gene Family Associated with Abiotic Stress in Rice
Source: Int J Mol Sci. 2024 May 18;25(10):5513. doi: 10.3390/ijms25105513 (PMC11121893; doi:10.3390/ijms25105513)
Supplement: Supplementary file 1 [file ijms-25-05513-s001.zip › ijms-2992677-supplementary.pdf]

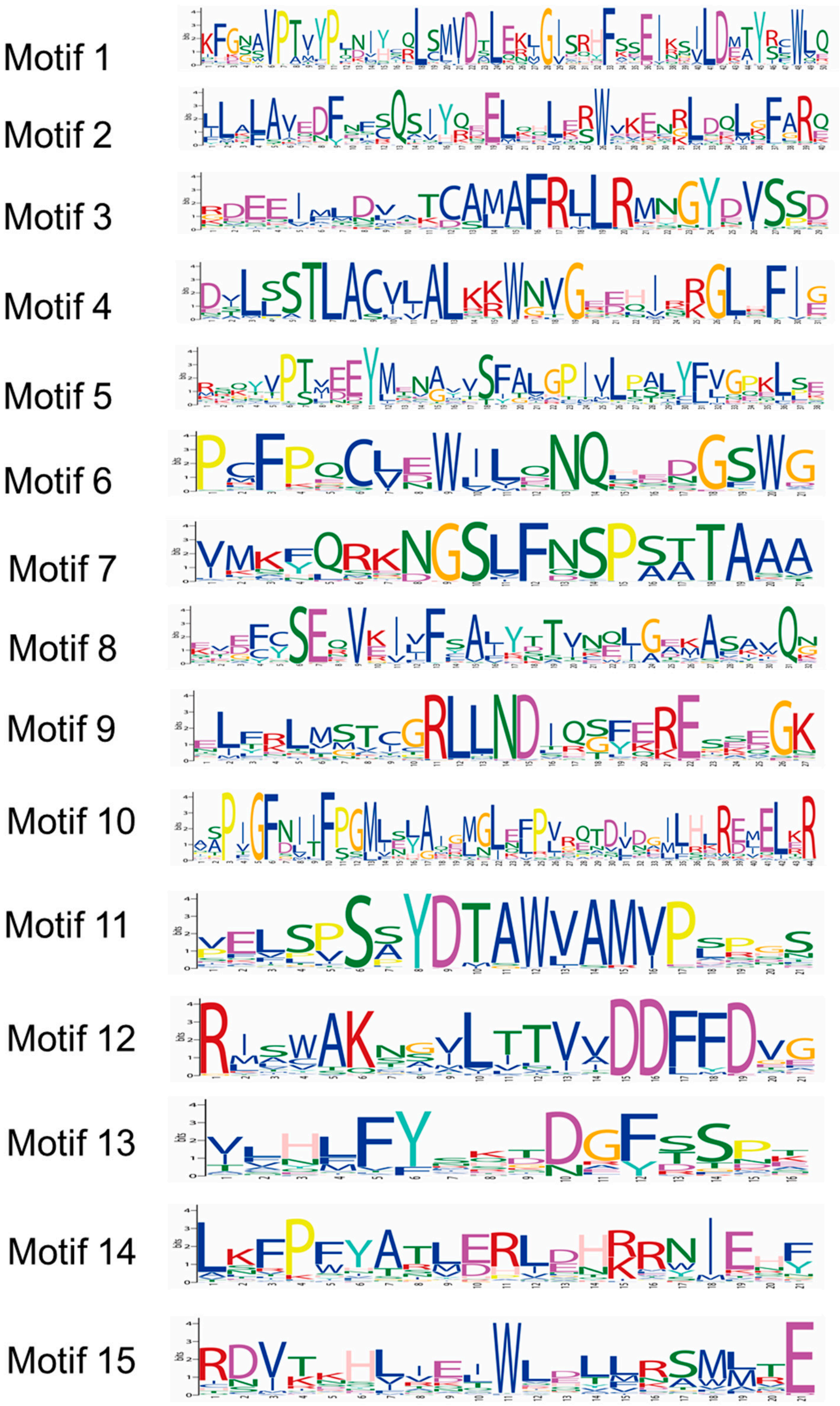

Figure S1 Representation of the Amino Acid Residue Sequences for the Identified 15 Motifs. Detailed sequences of the identified motifs are depicted as they appear in Figure 2A. Analysis of motif sequences was conducted utilizing the MEME Suite (https://meme-suite.org/meme/).

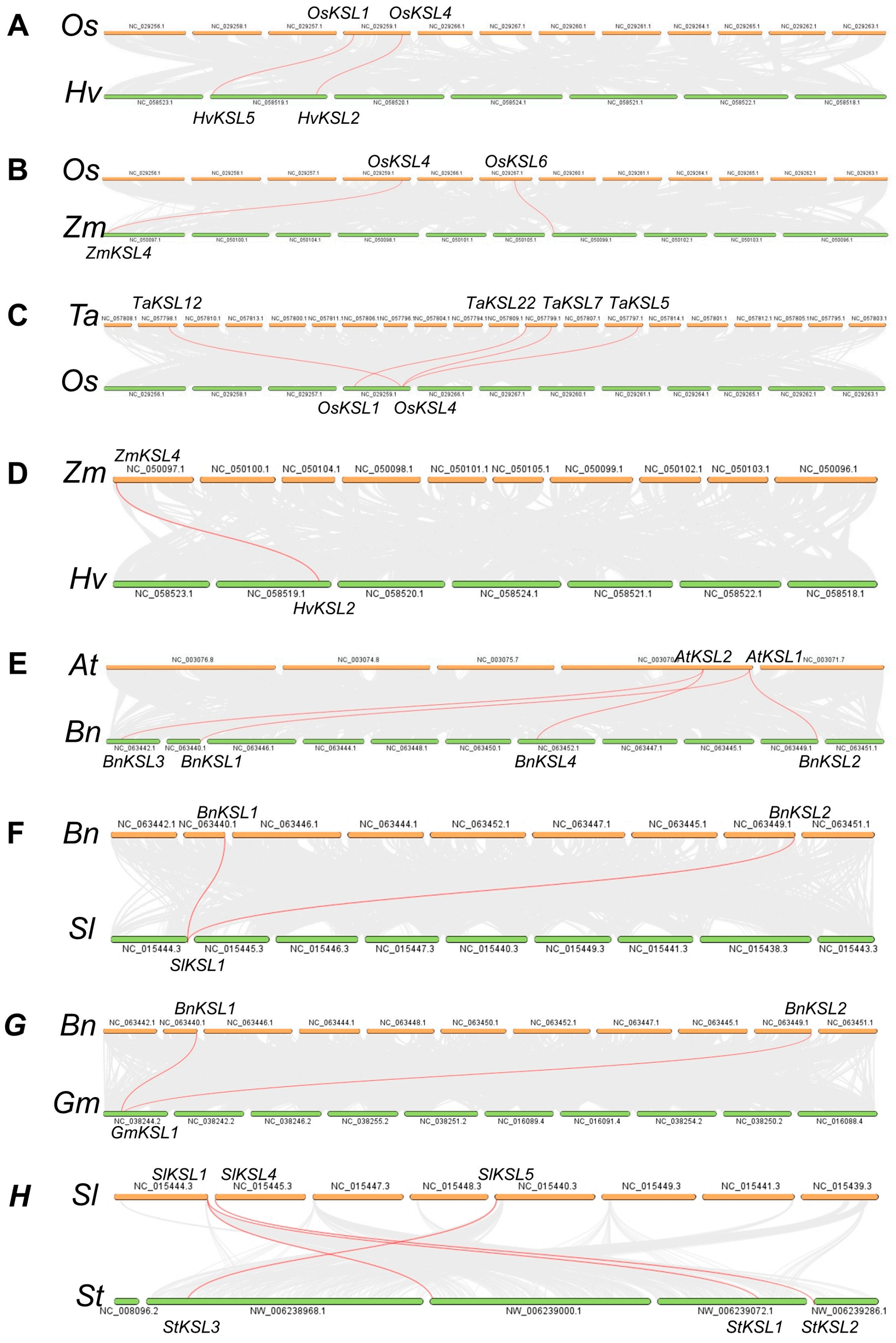

Figure S2 Collinearity analysis of *KSLs* gene across monocotyledonous and dicotyledonous species.

(A-D) the conservation of synteny for *KSL* genes within selected monocotyledonous species. *Hv*, *Hordeum vulgare*; *Os*, *Oryza sativa*; Ta, *Triticum aestivum*; Zm, *Zea may*.

(E-H) the conservation of synteny for *KSL* genes within selected dicotyledonous species. Sl, *Solanum lycopersicum*; St, *Solanum tuberosum*; Bn, *Brassica napus*; At, *Arabidopsis thaliana*.

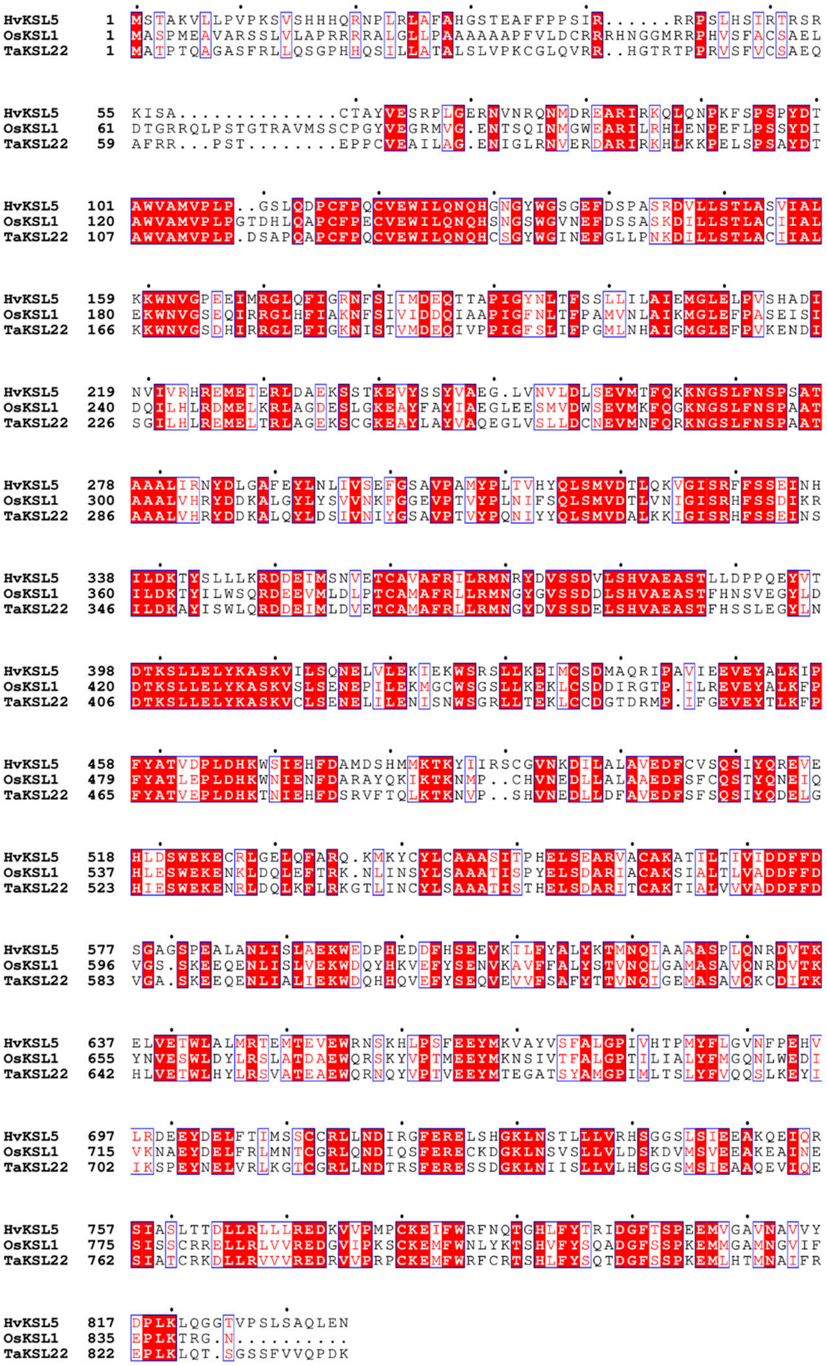

Figure S3 Protein sequences alignment of KSLs related to OsKSL1 in collinearity analysis. Residues in white characters on a red background denote fully conserved amino acids across the sequences. Red characters encased in blue boxes exhibit a high level of sequence similarity. The alignment underscores the conserved regions and similar motifs within the KSL family in relation to OsKSL1.

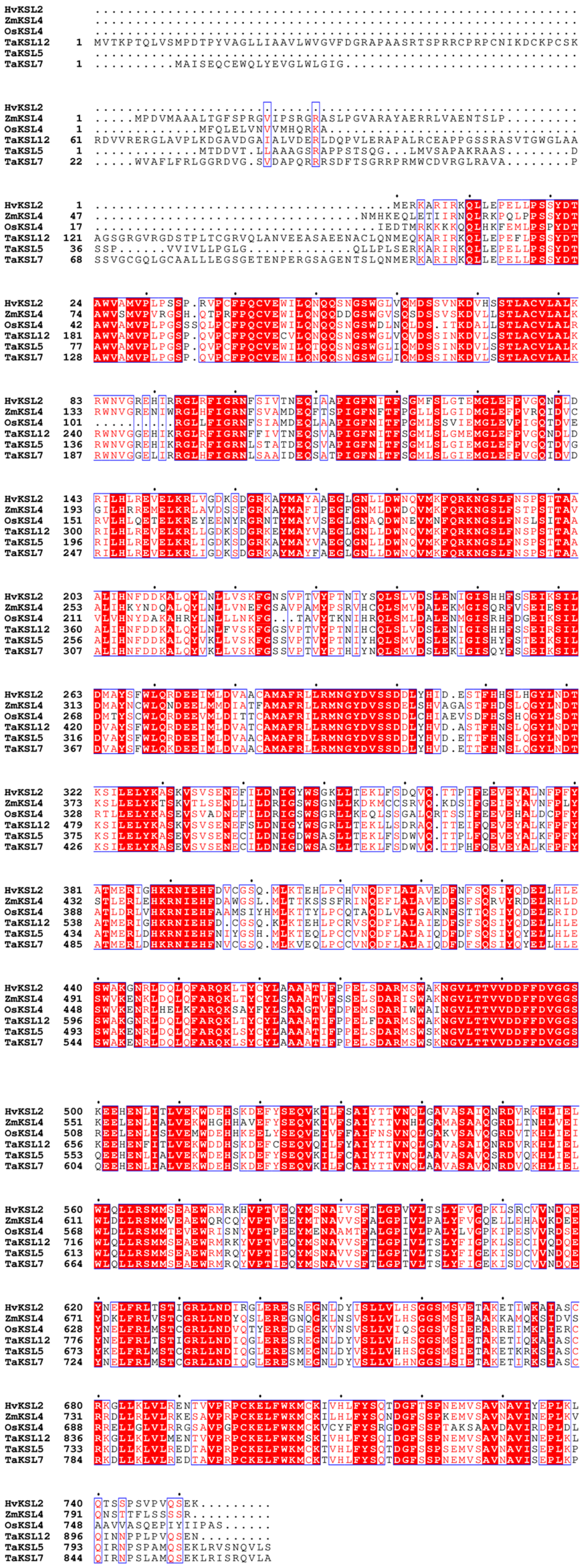

Figure S4 Protein sequences alignment of KSLs related to OsKSL4 in collinearity analysis. Residues depicted in white text on a red background denote identical amino acids across the aligned sequences. Those highlighted by red text within a blue box signify conserved residues exhibiting high similarity.

```
SlKSL4    1  MIIGCRSKIIIISHHKLGNGKTISSKDAIFQRSCRARCSHSTTSSMS......IRISFPI
StKSL2    1  MIVGYRSTIITLSHHKLGNGKTISSN.AIFQRSCRVRCSHSTTPSMDGFEAARERIRETF

SlKSL4   55  SQTRGDQIQYIISFVGLYS...................................LLEEFL
StKSL2   60  GKVELSPSSYDTAWVAMVPSKHSLNEPCFPQCLDWIIENQREDGSWGLKPSHPLLLKDSL

SlKSL4   80  ..................HFLSGLGFIETYGCAVDNKDQISPLGFEVIFSSMIKFAEKLN
StKSL2  120  SSTLACLLALSKWRVGDEQIKRGLGFIETYGWAVDNKDQISPLGFEIIFSSMIKFAEKLN

SlKSL4  122  LNLPLNLH.LVNLVNCKKDSTIKRNDEYMGEGVGELCDWKEIIKLHQRQNGSLFDSPATT
StKSL2  180  LNLPLDLDIIVNLVNCKRDSTIKRNDEYMGEGLGELCDWKEIIKLHQRQNGSLFDSPATT

SlKSL4  181  AAALIYHQRDQKCYEYLNSILQQHKNWVPTMYPTMIHSLLCLVDTLQNLGVHRHFKSEIK
StKSL2  240  AAALIYHQHDKKCYEYLNSILQKHKNWVPTMYPTKIHSLLCLVDTLQNLGVHRHFKSEIK

SlKSL4  241  KALDEIYRLWQQKNEEIFSNITHCAMAFRLLRMSNYDVSSDELAEFMDEEHFFTTSGKYT
StKSL2  300  EVLDEIYRLWQQKNEEIFSNVTHCAMAFRLLRMSNYDVSSDELAEFVVEEHFFATSGKYT

SlKSL4  301  SHVEILELHKASQLAIDQEKDDILDKINNWTRTFMEQKLLNNGFIDRMSKKEVELALRKF
StKSL2  360  SHVEILELHKASQLAIDHEKDDILDKINNWTTTFMEQNLLNNGFIDRMSKKEVELALRKF

SlKSL4  361  YTTYDRAENRRYIKSYEENNFKILKAAYRSPNINNKDLLIFSIHDFDLCQTQHREELQQL
StKSL2  420  YTTYDRTEYRRYIKSYEENNFKILKAAYRSPNINNKDLLIFSIHDFELCQAQHREELQQL

SlKSL4  421  KRWFQDCRLDQLGLSEQFISTTYLIGIAVVSEPEFSNARLMYAKYVMLLTIVDDLFDGFA
StKSL2  480  KRWFEDCRLDQLGLSEQLIYTSYLICVAIVTDIEFSDARLMYAKYVMLLTVVDDLFESFA

SlKSL4  481  SKDELLNIIQLVERWDDYASVGYNSERVKVFFSVFYKSIEELATIAEIKQGRSVKNHLIN
StKSL2  540  SEDELLNIIELVERWDDYASVGYNSERVKVFFSIFYKSIEELATIAEIKQGRSVKNHLTD

SlKSL4  541  LWLEVMKMMLIERIEWWTSKTIPSIEEYLYVTSITFGSRLIPLTTQYFLGIKISKDLLES
StKSL2  600  LWLEVMKWMLIERLEWWTSKTIPSIEEYLYVTSITFGSRLISLTTQYYLGIKISKDILES

SlKSL4  601  DEIYGLCNCTGIVMRLLNDLQTYKREQGESSMNLVTILMTQSPRRTNICEEEAIMKIKEI
StKSL2  660  DEIYGLWNCSGRVMRLLNDLQASKREQKEGSINLVTILMT......NISEEEAIMKIKEI

SlKSL4  661  LEMNRRELLKMVLVQKKGSQLPQLCKDIFWRTSKMVYFTYSHGDEYRFPEEMKNHIDEVI
StKSL2  714  LESNRRELLKMVLVQKKGSQLPQLCKEIFGRTSKMAYFTYSHGDEYRFPEEMKNHIDEVF

SlKSL4  721  YKPLNH
StKSL2  774  YKPLNH
```

Figure S5 Protein sequences alignment of SlKSL4 and StKSL2.

Residues depicted in white text on a red background denote identical amino acids across the aligned sequences. Those highlighted by red text within a blue box signify conserved residues exhibiting high similarity.

```
SlKSL5    1  MDSSPLSSIESLVHDIKKDLFSNQENFNTFLTKTTFAYDIAWLAMIPFDNQKKEANGPMF
StKSL3    1  MDSSSLSSIESLVHVIKKDLFSNQDNFNTFFTKTS.AYDIAWLAMIPFNNQEKEDNGPMF

SlKSL5   61  VSCLNWILNNQNEQGFWGESNGENQIPTINTLPITLACMVALKKWNVGERHIKKGLKFVH
StKSL3   60  ESCLNWILNNQNEQGFWGESNGEN.LPTINTLPITLACMVALKKWNVGERHIRKGLKFIH

SlKSL5  121  ANMYTLLKENSQCFPCNWFTIVFPSILQLAKAQGLEIIIVNGSNKLLSDVIMKKKALLES
StKSL3  119  ANMNTLLKENSQCFPC.WFTIVFPSIFQLAKAQGLEIIIVNGSNKLVSDVFMKKKALLES

SlKSL5  181  ENLNDNIVSE............AQAFMSTGNKKCLQYLMSIVQQCPNGVSSRFPVDEELK
StKSL3  178  GKLNDNIVNEEDGSLFQSPSSIAQAFMSSGNKKCLEYLMSIVQKCPNGVPSRFPVDEELK

SlKSL5  229  MLCMVDNIQRMGLAKHFEKEIEQILDQVYNNYQINNLKNNNEYLLSSEEEKTSDDIPDKL
StKSL3  238  MLCMVDNIQRMGLAKHFEKEIEQILDQVYNNYQINNSQNNNEYLLSSEEEKTSDDIAEKL

SlKSL5  289  LKDSLVFRLLRLQGYKINQGSFCWFLQ.PHLKDKIEKNKEQFTYVMYNVYRATDLMFNGE
StKSL3  298  LKDSLAFRLLRLQGYKINQGSFCWFLQQPQIRDKIEKNKKHFTYVMYNVYRATDLMFNGE

SlKSL5  348  SQMEEARFFARNFLMNNDNYLSLFPSLQKVIEHELNVPWFARLEHLDHRLWIELNQSIPL
StKSL3  358  SQIEEARSFARNFLVNNDN.LSLFPALQKVIEHELNVPWFARLEQLDHRLWIELNQSIPL

SlKSL5  408  SIGKSSFYWLSYLQNEKLMQLAVQNYEYRQSIYRKELEDLKRWSKEKGLVDIGFGREKTT
StKSL3  417  SIAKSSFYWLAYLQNEKLMQLAVQNYEYRQSIYRKELEDLKRWSKEKGLVDIGFGREKTT

SlKSL5  468  YSYFASAASSSSFLPYESFLRLIVAKCSIIITVADDFYDEEASLSELHILSEAIQRWDAK
StKSL3  477  YSYFASAASSSSFLPYESFLRLIVAKCSIVITVADDFYDEEASLSELHILSEAIQRWDAK

SlKSL5  528  NLDGPSKIIFEALDDLVCDVAKMYHLQHKIDITPQLRHMWKETFDAWMMESLWSRTSNLP
StKSL3  537  NLDGPSKIIFEALDDLVCDVAKMYHLQHKIDITPQLRHMWKETFDAWMMESLWSRTSNLP

SlKSL5  588  SRDKYLQVGMISVGAHILVLHAASLGCTILPNQVFGPINGQYENITKLLMTTTRLLNDIQ
StKSL3  597  SRDKYLQVGMISIGAHILVLHAASLGCSILPNQMFGPINGQYENITKLLMTTTRLLNDIQ

SlKSL5  648  SYQKEREVGKMNYTLLHLNENPRGQIEDSIDFVKVILGDKKKEFLENVLMDGFNDMPRTM
StKSL3  657  SYQKEREVGKMNYTLLHFNENPRGQIEDSIDFVKVILGDKKKEFLENVLMDGFNDMPRTM

SlKSL5  708  KLLHLSCLNVFHMFFNSCNLFDTKSAILEDIMRAIYIPLGQNEQTKVMAIQEKKIKKKIE
StKSL3  717  KLLHLSCLNVFHMFFNSCNLFDTKSAILEDIMRAIYIPLGQNEQTQIMPLQEKKKKKKIE

SlKSL5  768  IVKVNACVNYEINMKQLQVGVGISYVKRQNPKNYNSFGLSTKGFASHNLRLCFM
StKSL3  777  IIQVNACVNHEKNMKQ.QVGIGISFVKRQNPKNYNSFGWSTKGFVSHNLRLCFM
```

Figure S6 Protein sequences alignment of SlKSL5 and StKSL3.

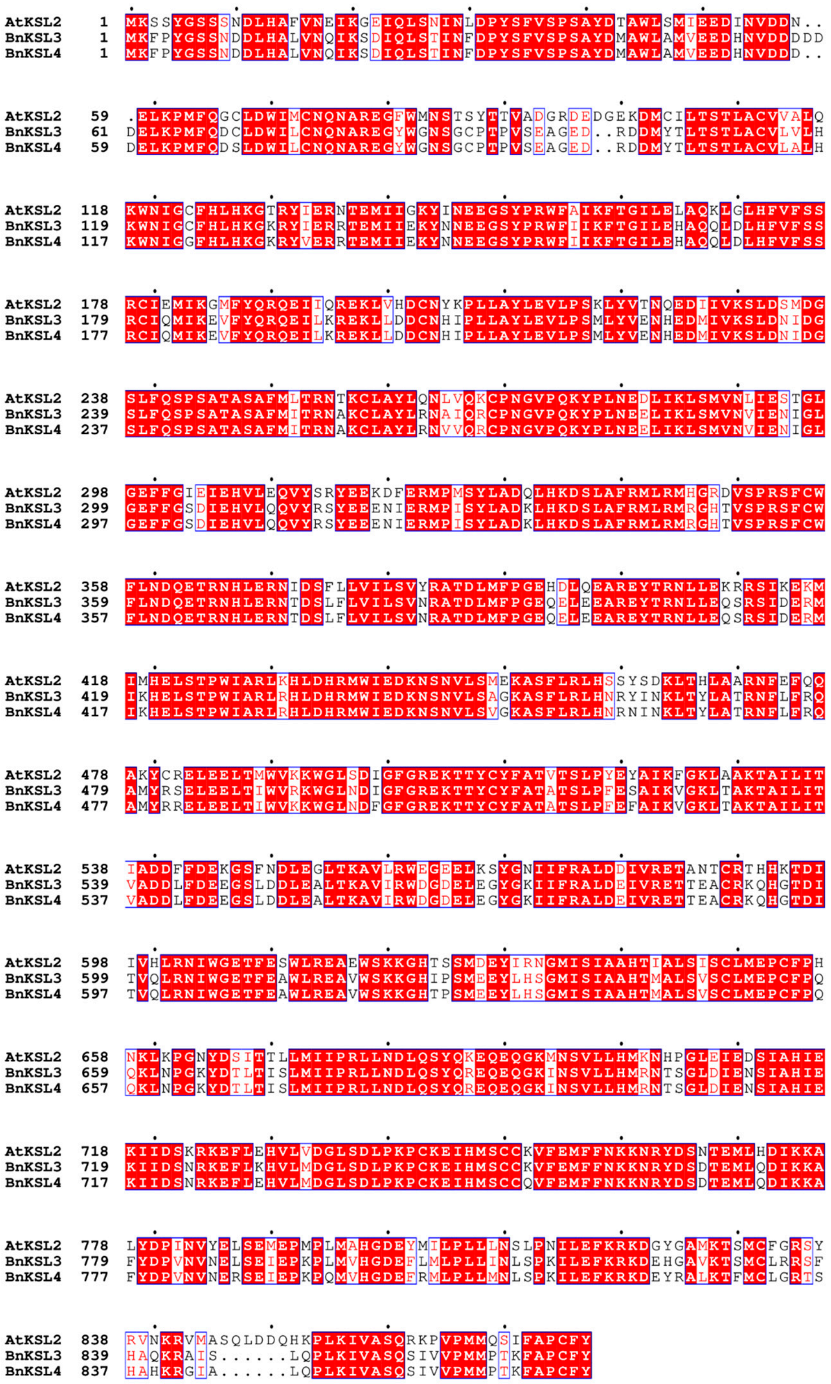

Figure S7 Protein sequences alignment of KSLs related to AtKSL2 pair in collinearity analysis.

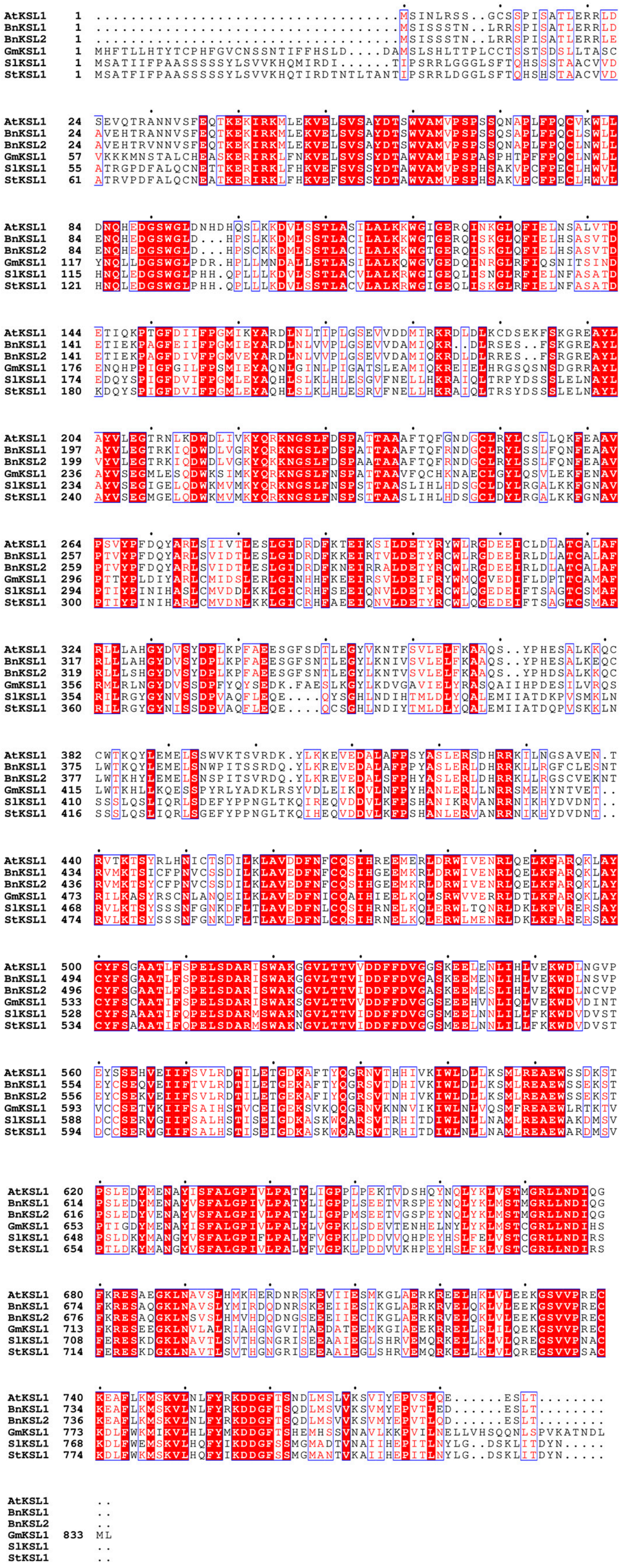

Figure S8 Protein sequences alignment of KSLs related to AtKSL1 pair in collinearity analysis.

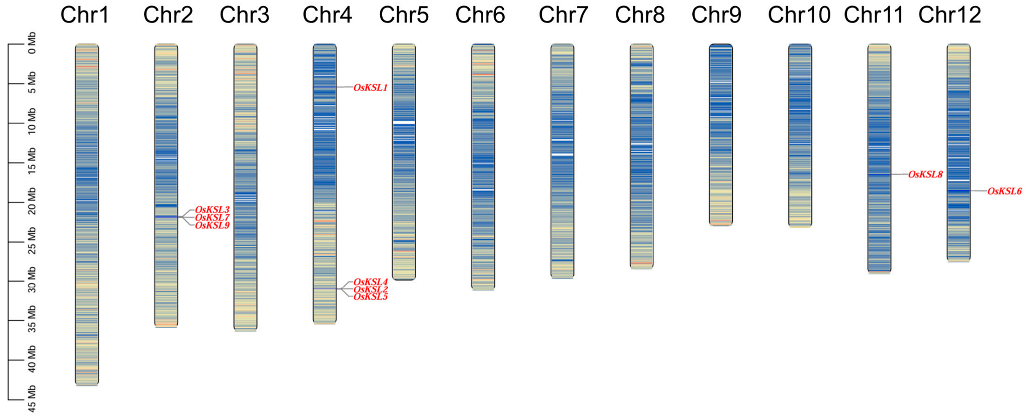

Figure S9 Chromosomal Localization of *OsKSL* Gene Members in *Oryza sativa*.

The diagram illustrates the positioning and relative distribution of *OsKSL* genes across the twelve rice chromosomes. Chromosome numbers are indicated at the top of each chromosome representation. A scale bar is provided on the left to denote the chromosomal length in megabase pairs (Mbp).

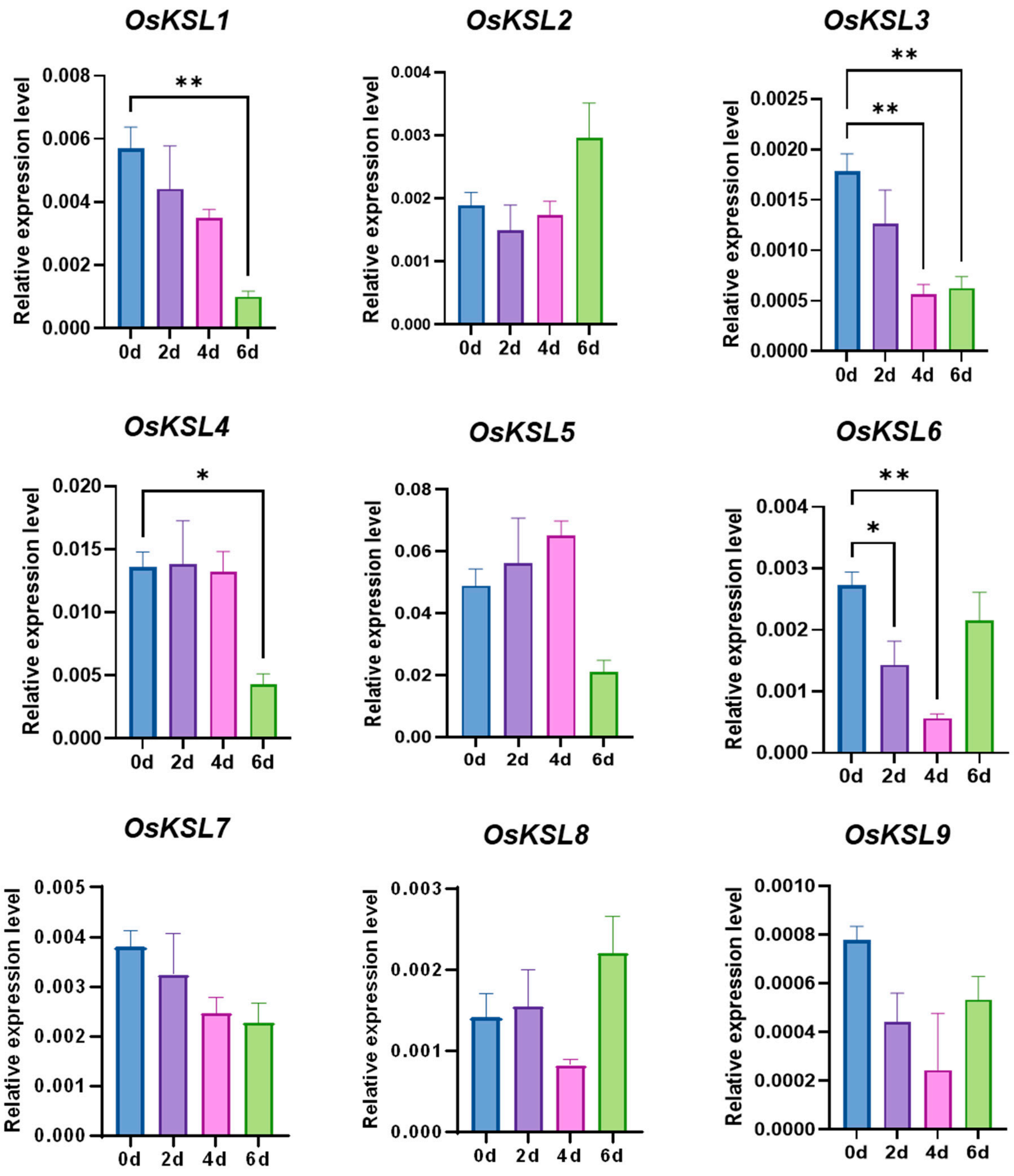

Figure S10 qRT–PCR analysis of *OsKSL* gene family in darkness

6 d-old seedlings grew without light for 6 days. Aerial tissues were sampled in description time points (0d, 2d, 4d, and 6d) (n = 4) for RNA extraction and qRT-PCR. * $P < 0.05$ and ** $P < 0.01$ (Student's *t*-test). Three biological replications were performed. Bar graphs show means. Error bars represent ± SE (n = 3). *OsUBQ6* was used as internal control.

Table S1 Plant genomes used in this study

Species	GenBank	RefSeq	Size (Mb)	WGS accession	Assembly
Selaginella moellendorffii	GCA_000143415.2	GCF_000143415.4	212.3	ADFJ01	V1.0
Arabidopsis thaliana	GCA_000001735.2	GCF_000001735.4	119.1	N/A	TAIR10.1
Brassica napus	GCA_020379485.1	GCF_020379485.1	1,001	JAGKQM01	Da-Ae
Phalaenopsis equestris	GCA_001263595.1	GCF_001263595.1	1,064	APLD01	ASM126359v1
Nicotiana tabacum	GCA_000715135.1	GCF_000715135.1	3,643	AYMY01	Ntab-TN90
Solanum lycopersicum	GCA_000188115.4	GCF_000188115.5	827.4	AEKE03	SL3.1
Solanum tuberosum	GCA_000226075.1	GCF_000226075.1	705.8	AEWC01	SolTub_3.0
Lactuca sativa	GCA_002870075.4	GCF_002870075.4	2,590	NBSK02	Lsat_Salinas_v11
Helianthus annuus	GCA_002127325.2	GCF_002127325.2	3,010	MNCJ02	HanXRQr2.0-SUNRISE
Glycine max	GCA_000004515.5	GCF_000004515.6	978.4	ACUP04	Glycine_max_v4.0
Pisum sativum	GCA_024323335.2	GCF_024323335.1	3,796	JAMSHJ01	CAAS_Psat_ZW6_1.0
Vigna radiata	GCA_000741045.2	GCF_000741045.1	463.1	JJMO01	Vradiata_ver6
Arachis hypogaea	GCA_003086295.2	GCF_003086295.2	2,557	PIVG01	arahy.Tifrunner.gnm1.KYV3
Gossypium hirsutum	GCA_007990345.1	GCF_007990345.1	2,305	VKGJ01	Gossypium_hirsutum_v2.1
Oryza sativa	GCA_001433935.1	GCF_001433935.1	373.8	N/A	IRGSP-1.0
Zea mays	GCA_902167145.1	GCF_902167145.1	2,182	CABHLF01	Zm-B73-REFERENCE-NAM-5.0
Triticum aestivum	GCA_018294505.1	GCF_018294505.1	14,567	JAGHKL01	IWGSC CS RefSeq v2.1
Hordeum vulgare	GCA_904849725.1	GCF_904849725.1	4,226	CAJHDD01	MorexV3_pseudomolecules_assembly
Sorghum bicolor	GCA_000003195.3	GCF_000003195.3	708.7	ABXC03	Sorghum_bicolor_NCBIv3

N/A: data was not available.

Table S2 The primers list

Primer	Sequence
OsKSL1-qpcr-F	CTCTCTGCTGCTGCCACCAT
OsKSL1-qpcr-R	GGCAACAAGTGTGAGCGCAA
OsKSL2-qpcr-F	GCGTGGCGGTCACAAGAGAT
OsKSL2-qpcr-R	CCTGATGTGCTCCTGCCCAA
OsKSL3-qpcr-F	CGCAAAGGCTTCCGCGTTAC
OsKSL3-qpcr-R	GGCAGATGCTGGCTCCTCTG
OsKSL4-qpcr-F	TCCATGCTTCCCACAATGCGT
OsKSL4-qpcr-R	GCCAGTGTGGACAAGAGAGCA
OsKSL5-qpcr-F	CCGGATGGCGATGGAGATGG
OsKSL5-qpcr-R	TTCCACCGTGGCAGATCACC
OsKSL6-qpcr-F	TTGGTGAGCTCAGCGAAGGC
OsKSL6-qpcr-R	GGCAAACGCTCCAGACACCT
OsKSL7-qpcr-F	CGAACACTATCGGCCGCCTT
OsKSL7-qpcr-R	CCATCGATGCCTCCGGTGAG
OsKSL8-qpcr-F	CAAGCGTATCGGCGCAAAGG
OsKSL8-qpcr-R	CTCTGCCTCGGTCAGCATGG
OsKSL9-qpcr-F	TGTTGACCGAGGCCGAATGG
OsKSL9-qpcr-R	ATGCCGCCGAGGTGATGATG
OsUBQ6-qpcr-F	TGCTATGTACGTCGCCATCCA
OsUBQ6-qpcr-R	AATGAGTAACCACGCTCCGTC
